# Improve the product structural robustness based on network motifs in product development

**DOI:** 10.1038/s41598-022-15056-2

**Published:** 2022-06-28

**Authors:** Yongbo Ni, Yingxia Ou, Yupeng Li, Na Zhang

**Affiliations:** Department of Industrial Engineering, China University of Mining and Technology, Xuzhou, 221116 Jiangsu China

**Keywords:** Engineering, Mechanical engineering

## Abstract

The stability and safety of products will be reduced if product structures are vulnerable to failures of key components. Existing methods for improving product structural robustness mainly focus on some key components, but they cannot provide designers with universal and explicit structure optimization strategies. From the viewpoint of product structural networks, the motif is the fundamental meta-structure, and it is efficient to analyse product structural properties. Motivated by this, strategies to improve product structural robustness are explored by considering relationships between typical motifs and product structural robustness. First, product structural networks are constructed by collecting the structural information of a series of product generations. Second, typical (anti-) motifs are identified based on an enumeration algorithm, and the robustness is measured considering the largest connected cluster. Then, relationships between the frequency of different motifs and product structural robustness are obtained through principal component regression. The results of a case study on the smartphone show that anti-motifs are negative for product structural robustness. Motifs with loop structures are positive for product structural robustness. Accordingly, relevant strategies to improve product structural robustness in product development are developed.

## Introduction

Networked systems are those that can be represented by complex networks, and they play a vital role in many scientific and engineering domains^1^. As common networked systems, products are always subjected to various failures in their lifetimes (e.g., the failure of components^2^, design changes derived from emerging customer requirements^3^, changed policies^4^, and advanced technology^5^). These failures can be generally sorted into two categories: failures of noncritical components and failures of key components (*FKC*)^6^. Product structural networks are highly robust under failures of noncritical components. However, they are extremely vulnerable under *FKC* due to their scale-free property^7^. For instance, product structures are not significantly affected by the failure of noncritical modules, but they will rapidly fragment when key modules (hubs) fail^8^. The vulnerability of product structures under *FKC* will reduce the stability and safety of products and increase the cost of maintenance and redesign^9,10^. Therefore, to keep the product structure stable and control cost in the product lifetime, it is necessary to improve product structural robustness under *FKC*.

Motifs are the simple building blocks of complex networks^11^. They have proven to be useful to describe the topological and functional properties of various networked systems^12^. For example, the structures and evolution characteristics of passenger airline networks are efficiently explored through the three-node and four-node motifs^13^. In addition, motifs are also beneficial for revealing answers to many important biological questions, e.g., the complex structure of gene systems^14^. Although products are common networked systems, their structural properties have not been well explored through motifs. In particular, the properties of product robustness are closely associated with the product structure. Existing studies on robustness by complex networks mainly focus on the effect of key nodes (components) or edges (relationship between components)^7,15^. They cannot offer universal and explicit structure optimization strategies for different network structures. With the changing network structures, designers need to spend considerable time reinventing the strategy to improve the network's robustness. As typical meta-structures of product structural networks, whether and how these different motifs affect product structural robustness have not been well revealed.

Motivated by these observations, a novel method to improve product structural robustness based on motifs is proposed. First, product structural networks are modeled by analysing the structural information of product generations. Then, for each product structural network, different (anti-) motifs are identified based on the enumeration algorithm, and the robustness is measured considering the largest connected cluster. Next, principal component regression (*PCR*) is adopted to uncover the relationship between the frequency of different motifs and the product structural robustness. Finally, to improve product structural robustness in product development, relevant strategies based on typical (anti-) motifs are proposed. The effects of eight typical motifs on the product structural robustness of smartphones are analysed to demonstrate the effectiveness of the proposed method.

This paper is organized as follows. The related literature is reviewed in "Literature review" Section. The proposed approach is introduced in "Methodology" Section. A case study is presented in "A case study" Section. The strategies and their implications are discussed in Discussion" Section. The conclusions and future works are summarized in "Conclusion and future work" Section.

## Literature review

In this section, two important issues, product robustness and network motifs, are reviewed.

### Analysis and improvement of robustness for products

Generally, robust/robustness design of products usually applies parameter design, tolerance design, and statistical analysis to reduce the sensitivity of product performance to noise factors^16,17^. However, research on the robustness of product structural networks in this study aims to analyse the ability of products to maintain normal structures or functions when suffering from failures. The studies that focus on the robustness of products/networked systems are divided into two categories: robustness analysis and robustness improvement^18^.

The robustness analysis aims at measuring the robustness, finding the collapse conditions, and revealing the evolution rules of robustness from the perspective of dynamics of network structure^19,20^. Many methods have been proposed to analyse the robustness based on different characteristics of networks. For example, Albert et al.^20^ measured the robustness of networks in terms of the “critical fraction” of nodes or links that failed to collapse a network. However, this definition of robustness cannot be well generalized in different real systems since the “critical fraction” can only be acquired until the network has completely collapsed. In addition, robustness has also been defined based on the graph spectrum^21^ or the shortest path^22^. However, these definitions are less intuitive for engineering applications. Focusing on the above issues, Schneider et al.^18^ defined robustness by considering the size of the largest components during all possible malicious failures. This measurement of robustness is simple and suitable in the entire process of the network gradually failing. Similarly, Mehrpouyan et al.^23^ analyse the robustness (resiliency) of the subsystem for the ramp system of an infantry fighting vehicle according to the largest group of components without failure. Meanwhile, Braha and Bar-Yam^7^ applied the degree-based strategy to analyse the robustness (ratio of normal nodes) of the product design network. In addition, Park and Kremer^19^ also analyse the robustness of product generations under random attacks and targeted attacks.

The robustness improvement focuses on enhancing product robustness by various methods to avoid complete collapse. The main approaches to improve robustness involve three strategies: protective strategy, recovery strategy, and structural optimization strategy. The protective strategy focuses on the preprotection or reinforcement of critical nodes. For example, Li et al.^8^ identified and protected the influential modules to enhance the product performance and stability. The recovery strategy aims at repairing the failed elements to ensure the system transitions to a safe state. For instance, Braha^24^ analysed the effect of different rates of failure and recovery on the robustness of product development when some design tasks failed. The structural optimization strategy concentrates on optimizing the topological structure of the products to improve their robustness. For example, the community structure is established for the mechanical product based on the network model. This product structure can enhance the independence between parts and reduce the scale of failure propagation^25^.

According to the aforementioned studies, there has been growing academic attention to the robustness analysis and improvement of products. However, existing studies mainly improve robustness by protecting or optimizing some key nodes/edges. They do not explore the robustness of products well from the perspective of meta-structure. For example, the analysis of the motifs can effectively uncover the system characteristics by some typical structures.

### Network motifs

Motifs are the basic construction of networked systems, and they describe the properties of systems from the perspective of meta-structure. Shen-Orr et al.^26^ first proposed the concept of the motif in analysing the transcriptional regulation network of Escherichia coli. They defined the meta-structures that recurred in the real network at frequencies much higher than those found in randomized networks as the “network motif”. Afterward, Milo et al.^11^ proposed the Z score to identify the motifs and applied the motifs to analyse the structural characteristics in biochemistry, neurobiology, and engineering domains. Thereafter, Baskerville and Paczuski^27^ sorted the different meta-structures into motifs and anti-motifs according to the Z score. They found that both motifs and anti-motifs are important to the structure of networked systems.

Motifs are widely applied in biology, sociology, and engineering. For example, Piraveenan et al.^28^ used four-node motifs to analyse the characteristics of metabolic networks. Xie et al.^29^ found that the three-node motif played an essential role in keeping communication stable in friendship networks. Furthermore, Milo et al.^11^ revealed the relationship between motifs and functions of logic electronic circuits. They found that motifs separated the circuits into two classes that directly correspond to the functional description.

Meanwhile, motifs are also beneficial for product development. Park and Kremer^19^ analysed the evolution characteristics of product structures through three-node and four-node motifs. They claimed that motifs could be used as a basic unit of a modular structure. Then, the modular structure (community structure)^30^ can efficiently reduce the impact of one failed module on other modules, which improves product structural robustness. In addition, modular structures are also beneficial for lowering manufacturing costs and enhancing the efficiency of research and development for products^31^. As the basis of the product modular structure, motifs have different types. However, how these different motifs impact product structural robustness has not been well explored. Whether the changes in motifs in product structure will affect product structural robustness should be further investigated. Meanwhile, since the scales of product structural networks are different, there is still a problem in reasonably comparing the change in the occurrence times for motifs in product structural networks with different sizes.

In summary, the contribution of this study is twofold as follows.

1. A novel analysis method for product structural robustness considering the motifs of product structural networks is proposed. Rather than only considering the improvement of some key nodes/edges, this method optimizes product structural robustness from the perspective of the meta-structure. Thus, it can provide designers with some universal and typical motif-based strategies to improve product structural robustness.
2. The definition of frequency for motifs is proposed, and the principal components regression model is established to effectively clarify the relationships between the frequency of different motifs and product structural robustness. The frequencies of motifs can eliminate the effect of different scales of product structural networks. *PCR* can reduce the multicollinearity between different motifs.

## Methodology

**Figure 1:**
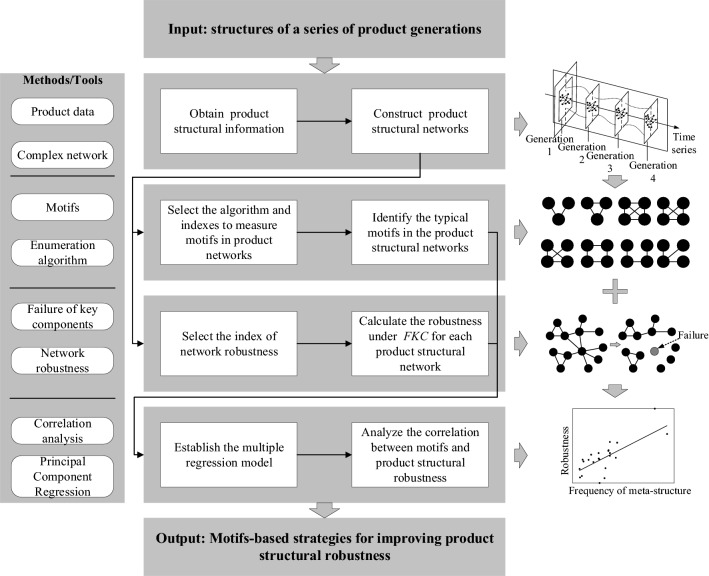
Technical framework of the proposed method.

The process of the method based on motifs to improve product structural robustness is shown in Fig. 1. Above all, structural networks of a series of product generations are constructed based on their structural information. Then, different (anti-) motifs are identified and product structural robustness is measured. Afterwards, a model of *PCR* analysis is established to analyse the relationships between product structural robustness and motifs. Finally, the strategies based on typical (anti-) motifs are proposed to improve product structural robustness.

### Construction of product structural networks

**Figure 2:**
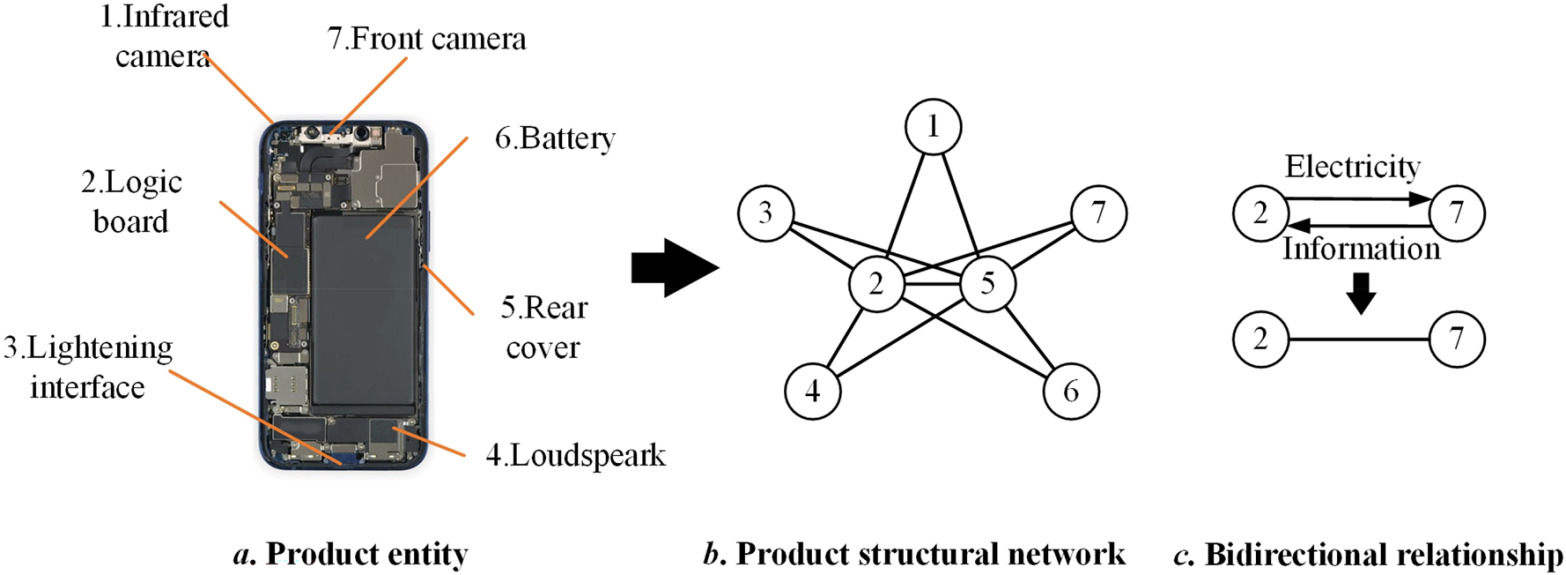
Structural network of a single product entity. (Adapted in part with free permission from iFixit, https://www.ifixit.com/Teardown/iPhone+12+and+12+Pro+Teardown/137669. Licenced under the CC BY open access licence).

**Figure 3:**
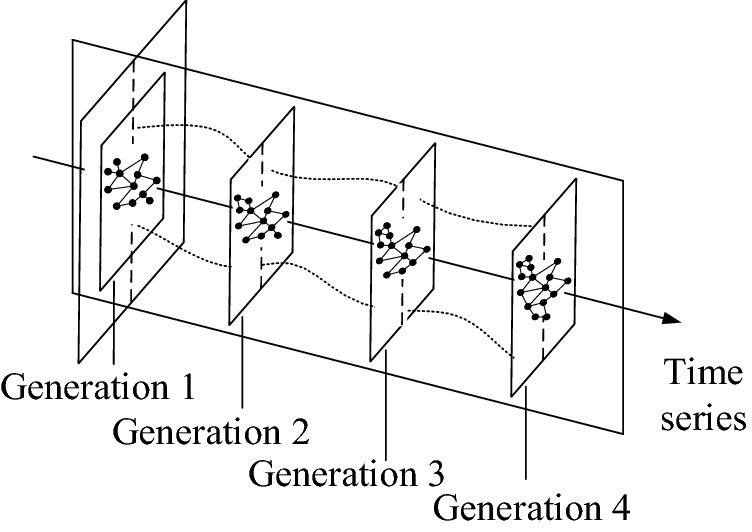
Illustration of product structural networks for product generations.

As shown in Fig. 2, in a single product structural network, nodes represent components in the product entity. Edges between nodes represent the relationship between components. Then, by collecting the information of a series of product generations, product structural networks can be acquired as shown in Fig. 3. The functional relationships (material flow, energy flow, and information flow)^32^ and structural (mechanical) relationships are both considered to model the product network. The functional relationships are directed, which consider the directions of different flows^32^. The structural relationships are usually regarded as undirected for the components connecting with each other^33^. In this study, the functional relationships between components are complex and intractable to be fully obtained. Meanwhile, functional relationships between many components are bidirectional. As shown in Fig. 2c, the electric energy transmits from the logic board to the camera, and the information of photos transmits from the camera to the logic board. Without considering the types of flows, the relationship between the camera and the logic board can be regarded as undirected. In addition, the product structure is the carrier of function realization and structural relationships directly affect the functional relationships. Consequently, the edges between nodes are simplified as undirected in this study. (Remarkably, there may be unidirectional flows between nodes in product structural networks. Therefore, the results may not completely reflect the characteristics of the product network.)

Each product structural network can be represented by an adjacency matrix **A** that contains *n* × *n* elements (*n* is the number of nodes in a network). The adjacency matrix is defined as

1 $$A_{ij} = \left\{ {\begin{array}{*{20}c} {1,} & {i{\text{f}} \;i \;{\text{and}}\; j \;{\text{are}} \;{\text{connected}}} \\ {0,} & {\text{otherwise }} \\ \end{array} } \right.$$

where *i* and *j* are the nodes in the network (*i* = 1, …, *n*, *j* = 1, …, *n*); and *A*_*ij*_ is the element of **A**.

### Identification of motifs and measurement of robustness for each product structural network

Based on product structural networks, typical motifs and robustness for each product generation can be obtained.

#### Identification of motifs based on the enumeration algorithm

In this study, motifs are defined as those meta-structures that appear more frequently in product structural networks than in random networks. Therefore, there are two steps to judge whether a meta-structure in product structural networks is a motif: *Step 1*: Counter the occurrence times of each meta-structure; and *Step 2*: compare the occurrence times of each meta-structure in product structural networks and that in the random networks.

**Figure 4:**
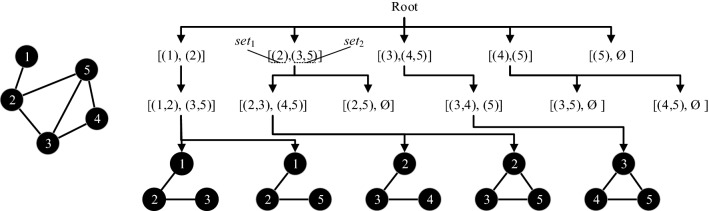
Detection of three-node meta-structures by enumeration algorithm.

The enumeration algorithm^34^ is employed to counter the occurrence times of each meta-structure. Figure 4 shows the process to identify the three-node meta-structures. Above all, select one node into *set*_1_ and add its neighbours into *set*_2_. (Remarkably, the labels of nodes in *set*_2_ must be larger than those of nodes in *se*t_1_. For example, if node 2 is selected into *set*_1_, then its neighbours (node 3 and node 5) are added to *set*_2_. However, node 1, which also connects to node 2, is not considered.) Then, move one node from *set*_2_ to *set*_1_ and update *set*_1_ and *set*_2_ (e.g., when node 3 is moved into *set*_1_, the *set*_2_ updates with node 4 and node 5). Finally, select one node from *set*_2_ and combine it with *set*_1_ to form the three-node meta-structure (node 2, node 3, and node 4 can form a V-shaped structure; node 2, node 3, and node 5 can form a loop structure). According to this process, the occurrence times of different meta-structures can be obtained.

Then, to efficiently compare the occurrence times of each meta-structure in product structural networks and random networks, the Z score index is adapted^11^.

2 $$Z_{q} = \frac{{N_{{real_{q} }} - \overline{N}_{{rand_{q} }} }}{{\sigma_{{rand_{q} }} }},$$

where *Z*_*q*_ is the Z score for the meta-structure *M*_*q*_ in product structural networks; ${\text{N}}_{{\text{real}}_{\text{q}}}$ is the occurrence times of *M*_*q*_;$\bar{N}_{{{\text{rand}}_{{\text{q}}} }}$ is the mean of occurrence times of *M*_*q*_ in the random networks; and $\sigma _{{rand_{q} }}$ is the related standard deviation. The number of each motif in the random networks follows a normal distribution. Generally, if *Z*_*q*_ > 2, *M*_*q*_ is a motif; otherwise, *M*_*q*_ is an anti-motif^27^.

As shown in Table 1, eight typical three-node and four-node (anti-) motifs are selected in this study to uncover the relationships between motifs and product structural robustness. There are three main reasons for choosing the eight motifs. First, three-node and four-node (anti-) motifs widely exist in product systems and play a vital role in product design and manufacturing^19,24^. Second, the number of multinode motifs is much smaller than the number of three-node and four-node motifs^27^. Thirdly, the multinode structures can also be formed by the eight basic three-node and four-node (anti-) motifs^27^.

**Table 1:** Three-node and four-node (anti-) motifs.

Types								
No	*M*_1_	*M*_2_	*M*_3_	*M*_4_	*M*_5_	*M*_6_	*M*_7_	*M*_8_
*Max*_*q*_	${\text{3C}}_{\text{n}}^{3}$	${\text{C}}_{\text{n}}^{3}$	${\text{C}}_{\text{n}}^{4}$	${\text{6C}}_{\text{n}}^{4}$	${\text{12C}}_{\text{n}}^{4}$	${\text{3C}}_{\text{n}}^{4}$	${\text{6C}}_{\text{n}}^{4}$	${\text{4C}}_{\text{n}}^{4}$

*Max*_*q*_ is the maximum number of occurrences for (anti-) motif *M*_*q*_ in a product structural network.

Because the sizes of networks vary, different product structural networks will have a different number of (anti-) motifs. To eliminate the effect of network size on motifs, the frequency of the (anti-) motif is defined as

3 $${\text{f}}_{\text{q}}\text{=}\frac{{\text{N}}_{{\text{real}}_{\text{q}}}}{{\text{Max}}_{\text{q}}}$$

**Figure 5:**
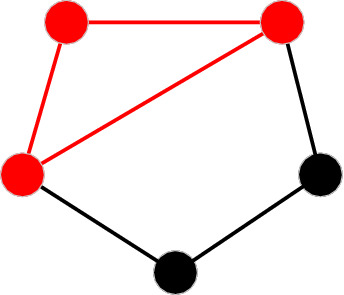
Motif of *M*_2_ in a simple network.

For example, there is only one *M*_2_ in the network in Fig. 5. The maximum number of occurrences for *M*_2_ is ${\text{C}}_{5}^{3}= \text{10}$. Then *f*_2_ in the network is 1/10 = 0.1. The maximum number of occurrences for each (anti-) motif under different network sizes (*n*) is listed in Table 1.

#### Measurement of robustness under failures of key components

**Figure 6:**
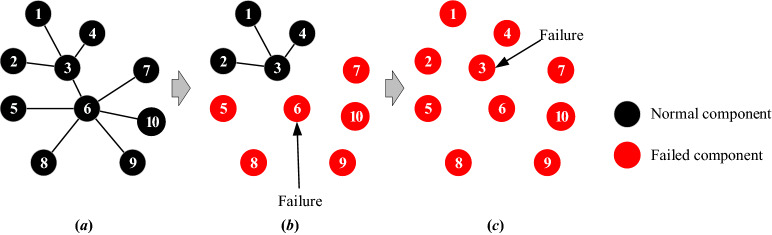
*FKC* of the network.

As mentioned in "Analysis and improvement of robustness for products" Section, the definition of robustness proposed by Schneider et al.^18^ is simple and suitable in the entire process of the gradually failing network. Therefore, it is selected to measure product structural robustness in this study. When a fault occurs, some nodes in product structural networks cannot work, and the ratio of the normal nodes shows the robustness of the product. The definition of product structural robustness can show the ability of a product to keep normal structures/functions. Product structural robustness (*φ*) is defined as

4 $$\varphi\;{{ = }}\;\frac{1}{{{n}}}\sum\limits_{{{{Q}} = {{1}}}}^{{{n}}} {{{s(Q)}}}$$

where *n* is the number of nodes; *Q* is the number of nodes that fail; and *s*(*Q*) is the fraction of nodes in the largest connected cluster after *Q* nodes fail^18^. As shown in Fig. 6b, when node 6 fails in the network, node 5, node 7, node 8, node 9, and node 10 fail too. Only four nodes can keep the normal structure/function. Therefore, *s*(*Q*) = 4/10 = 0.4.

For the pattern of failure, the degree-based strategy is employed in this study to analyse the product robustness under the intentional failure of key components. The degree-based strategy is widely applied in the failure simulation of nodes, and the degree of the node can represent the importance of the node in the network^18,23^. The nodes fail in sequence according to their node degree. The node degree is

5 $${\text{k}}_{\text{i}}\text{=}\sum_{{\text{j}}= \text{1} }^{\text{n}}{{\text{A}}}_{\text{ij}}$$

where *k*_*i*_ is the degree for node *i*. Taking the nodes in Fig. 6 as an example, the degrees for each node are *k*_1_ = *k*_2_ = *k*_4_ = *k*_5_ = *k*_6_ = *k*_7_ = *k*_8_ = *k*_9_ = *k*_10_ = 1, *k*_3_ = 4, and *k*_6_ = 6. Therefore, node 6 is the first to fail, followed by node 3, and then the other nodes fail. Figure 6b,c show that even if only two nodes (node 6 and node 3) fail, the whole network is affected.

### Obtain relationships between (anti-) motifs and product structural robustness

A multiple regression model is established to analyse the relationship between the predetermined eight motifs and product structural robustness. In the model, the frequency of each (anti-) motif is the independent variable and product structural robustness is the dependent variable. Since the formations of some (anti-) motifs in product structural networks are similar, *PCR*^35^ is employed to reduce the multicollinearity between independent variables.

The main processes of *PCR* for analysing the relationships between (anti-) motifs and product structural robustness include four steps.

#### Step 1: Correlation analysis

In this step, the correlation matrix is applied to analyse whether there is a relationship between the independent variable and dependent variable. The elements in the correlation matrix are defined as

6 $${\text{r}}_{\text{xy}}{=}\frac{{\text{S}}_{\text{xy}}}{{\text{S}}_{\text{x}}{{\text{S}}}_{\text{y}}}$$

where *r*_*xy*_ is the correlation coefficient. *r*_*xy*_ > 0, sample *x* and sample *y* are positively correlated; and *r*_*xy*_ < 0, *x* and *y* are negatively correlated. *S*_*x*_ and *S*_*y*_ are the standard deviations of the samples *x* and *y*. In this study, the samples are the robustness of product structural networks and the frequency of motifs. *S*_*xy*_ is the covariance between sample *x* and sample *y*. *Sxy* can be calculated as

7 $${\text{S}}_{\text{xy}}{=}\frac{{\sum }_{{\text{i}}= 1}^{\text{g}}\left({\text{x}}_{\text{i}}-\stackrel{\mathrm{-}}{\text{x}}\right)\left({\text{y}}_{\text{i}}-\stackrel{\mathrm{-}}{\text{y}}\right)}{{\text{g}}-1}$$

where *g* is the number of elements in the samples, *i* = 1, 2, …, *g*. $\stackrel{\mathrm{-}}{\text{x}}$ and $\stackrel{\mathrm{-}}{\text{y}}$ are the average values of sample *x* and sample *y*, respectively.

#### Step 2: Principal component analysis

The principal components (**C**) for the eight kinds of independent variables are obtained according to Eq. (8).

8 $${\mathbf{C}} = {\mathbf{a}} \cdot {\mathbf{f}}^{^{\prime}} ,$$

where **a** is the unit matrix corresponding to the eigenvalues of the correlation matrix^35^; and **f**^***'***^ is the normalized form of **f**, which is constructed of *f*_*rq*_ (the frequency of the *q*^*th*^ motif in the *r*^*th*^ product structural network). Then the elements in **f**^***'***^ can be computed by

9 $${{f}}_{{rq}}^{^{\prime}}{=}\frac{{{f}}_{{rq}}-\overline{{{f} }_{{q}}}}{{{S}}_{{q}}}$$

where $\overline{{{f} }_{{q}}}$ is the mean of *f*_*rq*_ and *S*_*q*_ is the related standard deviation.

#### Step 3: Regression analysis for the principal components

The relationships between product structural robustness (*φ*) and principal components are analysed through the regression model, as shown in Eq. (10). In this model, *α* is a constant term, and **β**^***'***^ is the matrix of the regression coefficients for the principal components. *ɛ* is the residual term.

10 $$\varphi = \alpha + {{\varvec{\upbeta}}}^{\prime } \cdot {\mathbf{C}} + \varepsilon$$

#### Step 4: Regression analysis for the independent variables

The regression model obtained in the last step concerns the principal components. To directly reflect the effect of each (anti-) motif on product structural robustness, the principal components should be converted to independent variables. According to Eqs. (8) and (10), the relationships between motifs and product structural robustness can be expressed as

11 $$\varphi = \alpha + {{\varvec{\upbeta}}} \cdot {\mathbf{f}}^{^{\prime}} + \varepsilon$$

where **β** is the matrix of the regression coefficient for the standardized independent variables, and **β = β**^***'***^·**a**.

### Motif-based strategies to improve product structural robustness

Based on the relationship between (anti-) motifs and product structural robustness, three possible motif-based strategies can be developed to improve product structural robustness.

- *Strategy 1* Increase the frequencies of (anti-) motifs that have a positive regression coefficient with product structural robustness. The (anti-) motif can improve product structural robustness when the regression coefficient between them is positive.
- *Strategy 2* Reduce the frequencies of (anti-) motifs that have a negative regression coefficient with product structural robustness. The (anti-) motif can decrease product structural robustness when the regression coefficient between them is negative.
- *Strategy 3* Protecting the key components and fragile components in (anti-) motifs. Some (anti-) motifs are essential for realizing product function, so protective measures are necessary to keep them working.

## A case study

**Figure 7:**
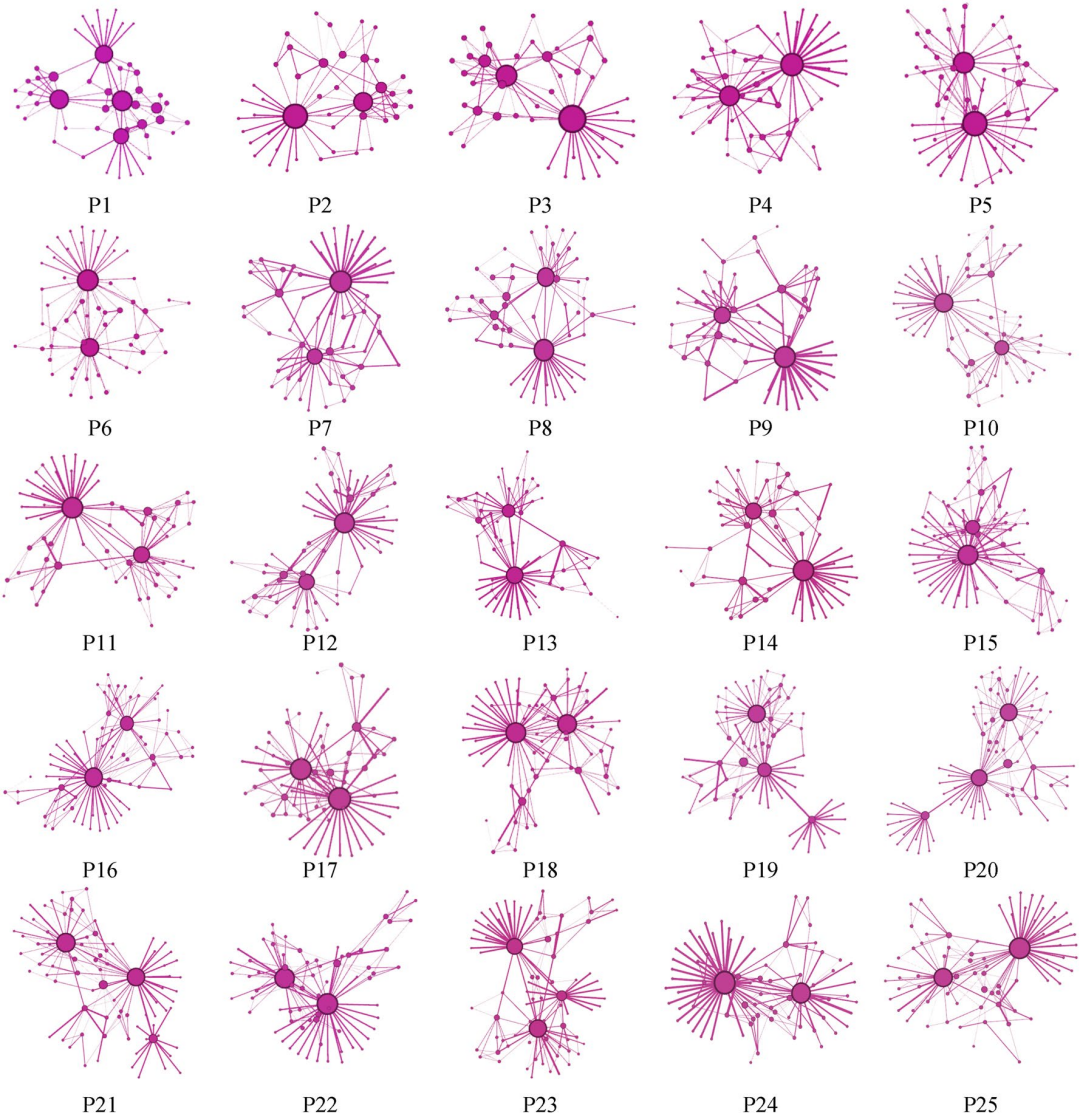
Product structural networks of the 25 generations of smartphones.

Smartphones are quickly evolving and their structures are easy to identify. Thus, the relationships between (anti-) motifs and the product structural robustness of 25 generations of smartphones ranging from June 2007 to October 2020 are analysed to demonstrate the proposed method. First, the product data of 25 generations of smartphones are collected from the iFixit website^36^. iFixit provides detailed information about the teardown of smartphones. Usually, a smartphone consists of six main parts (screen assembly, camera, logic board, buttons, battery, and cover), involving more than 50 components. (The relationship between components of each product is provided in the Supplementary information file). Then, based on the network model proposed in "Construction of product structural networks" Section, the components are represented as nodes and the relationships between components are represented as edges to form the product networks. As shown in Fig. 7, the size of each node directly relies on the node degree in the product networks. The specific number of nodes and edges for each generation is listed in Table 2.

**Table 2:** Statistics of nodes and edges of the 25 generations of smartphones.

*No*	Node	Edge	*No*	Node	Edge	*No*	Node	Edge
P1	47	90	P10	64	113	P19	72	128
P2	44	81	P11	67	117	P20	78	133
P3	44	81	P12	64	115	P21	81	138
P4	55	99	P13	64	115	P22	71	131
P5	56	99	P14	59	103	P23	79	138
P6	56	100	P15	68	121	P24	74	127
P7	58	102	P16	75	135	P25	75	132
P8	57	100	P17	69	123			
P9	59	102	P18	71	129			

### Motifs and anti-motifs in product structural networks

**Figure 8:**
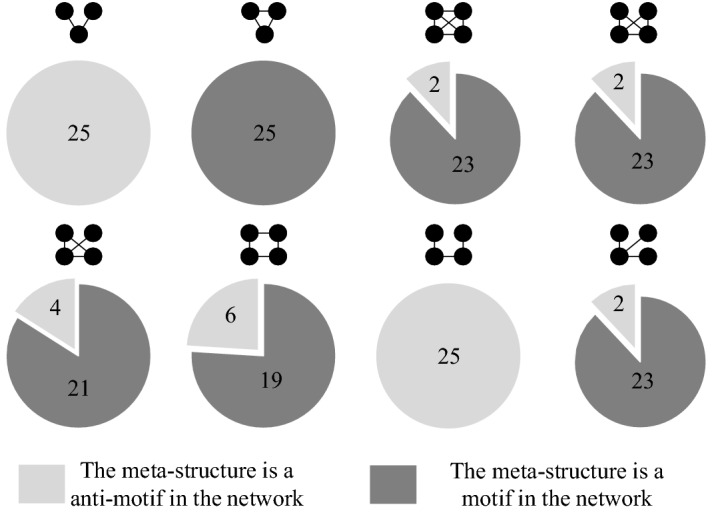
Motifs and anti-motifs of the 25 product structural networks.

The occurrence number of each meta-structure in both product structural networks and random networks is countered through the enumeration algorithm proposed in “Identification of motifs based on the enumeration algorithm” section. Next, the Z score of each motif in product structural networks is analysed based on Eq. (2). According to the value of the Z score (whether Z score > 2 or not), the types of each meta-structure are distinguished. As shown in Fig. 8, the meta-structures of *M*_1_ and *M*_7_ are always anti-motifs in the 25 generations of product structural networks. In contrast, the meta-structure of *M*_2_ is always the motif. In addition, the meta-structures of *M*_3_, *M*_4_, *M*_5_, *M*_6_, and *M*_8_ all have a high probability of being motifs and a low probability of being anti-motifs. For example, *M*_3_ has a 92% probability of being a motif in the 25 product structural networks. Thus, the meta-structures of *M*_2,_ *M*_3_, *M*_4_, *M*_5_, *M*_6_, and *M*_8_ are regarded as motifs from the global perspective. The meta-structures of *M*_1_ and *M*_7_ are regarded as the anti-motif.

As shown in Table 3, the number of each motif and anti-motif is countered. Then their frequencies are calculated according to Eq. (2), as shown in Table 4.

**Table 3:** Number of each (anti-) motif in the 25 product structural networks.

Types								
P1	39	328	3	55	458	22	724	1213
P2	36	308	3	80	487	16	635	1453
P3	36	308	3	80	487	16	635	1453
P4	38	578	7	48	1009	30	1189	2472
P5	34	597	4	48	972	26	1873	4190
P6	35	678	4	50	975	23	1844	4931
P7	39	801	3	50	656	17	2343	5551
P8	37	701	4	54	537	23	2236	4028
P9	40	754	3	59	620	16	3012	4768
P10	47	987	3	70	955	23	3954	7787
P11	48	1100	3	72	1042	23	3917	9527
P12	48	954	3	77	912	26	3703	7067
P13	48	954	3	77	912	26	3703	7067
P14	46	835	7	69	794	12	2895	5211
P15	49	1157	2	96	1191	23	5179	9760
P16	57	1507	2	131	1647	28	4910	15,392
P17	52	1249	2	117	1401	17	4712	10,819
P18	56	1331	2	135	1570	23	4802	11,788
P19	43	1344	1	146	1161	77	5140	12,987
P20	42	1475	1	145	1212	77	5549	15,864
P21	43	1794	0	145	1377	65	5738	17,276
P22	45	1453	1	114	1395	63	5763	11,882
P23	39	1703	1	74	1054	75	7235	18,740
P24	37	1591	1	119	1424	100	4934	16,449
P25	40	1766	5	139	1555	126	7164	17,780

**Table 4:** Frequencies of each (anti-) motif in the 25 product structural networks.

Types								
P1	6.7E-03	2.4E-03	1.7E-05	5.1E-05	2.1E-04	2.1E-05	3.4E-04	1.7E-03
P2	7.8E-03	2.7E-03	2.2E-05	9.8E-05	3.0E-04	2.0E-05	3.9E-04	2.7E-03
P3	7.8E-03	2.7E-03	2.2E-05	9.8E-05	3.0E-04	2.0E-05	3.9E-04	2.7E-03
P4	7.3E-03	1.4E-03	2.1E-05	2.3E-05	2.5E-04	1.5E-05	2.9E-04	1.8E-03
P5	7.2E-03	1.2E-03	1.1E-05	2.2E-05	2.2E-04	1.2E-05	4.2E-04	2.9E-03
P6	8.2E-03	1.3E-03	1.1E-05	2.3E-05	2.2E-04	1.0E-05	4.2E-04	3.4E-03
P7	8.7E-03	1.3E-03	7.1E-06	2.0E-05	1.3E-04	6.7E-06	4.6E-04	3.3E-03
P8	8.0E-03	1.3E-03	1.0E-05	2.3E-05	1.1E-04	9.7E-06	4.7E-04	2.5E-03
P9	7.7E-03	1.2E-03	6.6E-06	2.2E-05	1.1E-04	5.9E-06	5.5E-04	2.6E-03
P10	7.9E-03	1.1E-03	4.7E-06	1.8E-05	1.3E-04	6.0E-06	5.2E-04	3.1E-03
P11	7.7E-03	1.0E-03	3.9E-06	1.6E-05	1.1E-04	5.0E-06	4.3E-04	3.1E-03
P12	7.6E-03	1.2E-03	4.7E-06	2.0E-05	1.2E-04	6.8E-06	4.9E-04	2.8E-03
P13	7.6E-03	1.2E-03	4.7E-06	2.0E-05	1.2E-04	6.8E-06	4.9E-04	2.8E-03
P14	8.6E-03	1.4E-03	1.5E-05	2.5E-05	1.5E-04	4.4E-06	5.3E-04	2.9E-03
P15	7.7E-03	9.8E-04	2.5E-06	2.0E-05	1.2E-04	4.7E-06	5.3E-04	3.0E-03
P16	7.4E-03	8.4E-04	1.6E-06	1.8E-05	1.1E-04	3.8E-06	3.4E-04	3.2E-03
P17	7.9E-03	9.9E-04	2.3E-06	2.3E-05	1.4E-04	3.3E-06	4.5E-04	3.1E-03
P18	7.8E-03	9.8E-04	2.1E-06	2.3E-05	1.3E-04	3.9E-06	4.1E-04	3.0E-03
P19	7.5E-03	7.2E-04	9.7E-07	2.4E-05	9.4E-05	1.2E-05	4.2E-04	3.2E-03
P20	6.5E-03	5.5E-04	7.0E-07	1.7E-05	7.1E-05	9.0E-06	3.2E-04	2.8E-03
P21	7.0E-03	5.0E-04	0.0E + 00	1.5E-05	6.9E-05	6.5E-06	2.9E-04	2.6E-03
P22	8.5E-03	7.9E-04	1.0E-06	2.0E-05	1.2E-04	1.1E-05	4.9E-04	3.1E-03
P23	7.2E-03	4.9E-04	6.7E-07	8.2E-06	5.8E-05	8.3E-06	4.0E-04	3.1E-03
P24	8.2E-03	5.7E-04	8.7E-07	1.7E-05	1.0E-04	1.4E-05	3.6E-04	3.6E-03
P25	8.7E-03	5.9E-04	4.2E-06	1.9E-05	1.1E-04	1.7E-05	4.9E-04	3.7E-03

### Robustness of product structural networks

**Figure 9:**
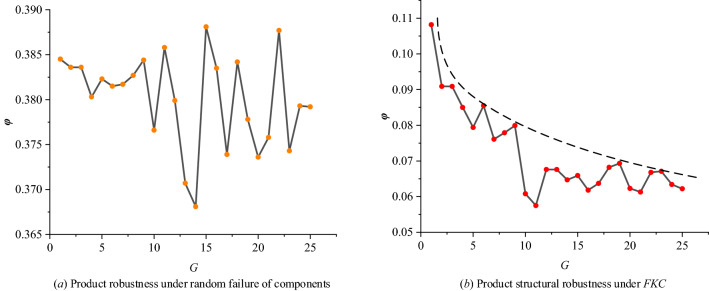
Product structural robustness under random attacks and *FKC.*

As shown in Table 5, the robustness for the 25 generations of product structural networks is obtained according to Eq. (4). As shown in Fig. 9, product structural robustness under the random failure of components is more than 3 times the robustness under *FKC*. In addition, the robustness under random attacks for the product generations fluctuates in the interval of [0.365, 0.390]. However, the robustness under *FKC* is smaller than 0.12. With the evolution of the product structure, it gradually decreases. This is caused by the increasing degree of internal integration of smartphones. With the failure of the key components, many more components connected with them have a high probability of failure. Remarkably, due to the progress and maturity of technology, the performance of components is improving, and the probability of failures of the components will be reduced. This study only considers the situation after *FKC* but does not aim at whether the key components will fail.

**Table 5:** Robustness of the 25 generations of product structural networks.

*No*	*φ*	*No*	*φ*	*No*	*φ*	*No*	*φ*	*No*	*φ*
P1	0.1132	P6	0.0772	P11	0.0526	P16	0.0548	P21	0.0479
P2	0.079	P7	0.0687	P12	0.054	P17	0.0555	P22	0.054
P3	0.079	P8	0.0736	P13	0.054	P18	0.0565	P23	0.0521
P4	0.0717	P9	0.0681	P14	0.0603	P19	0.0565	P24	0.0517
P5	0.0676	P10	0.0562	P15	0.0606	P20	0.05	P25	0.0519

### Analysis of relationships between motifs and product structural robustness based on principal component regression

**Figure 10:**
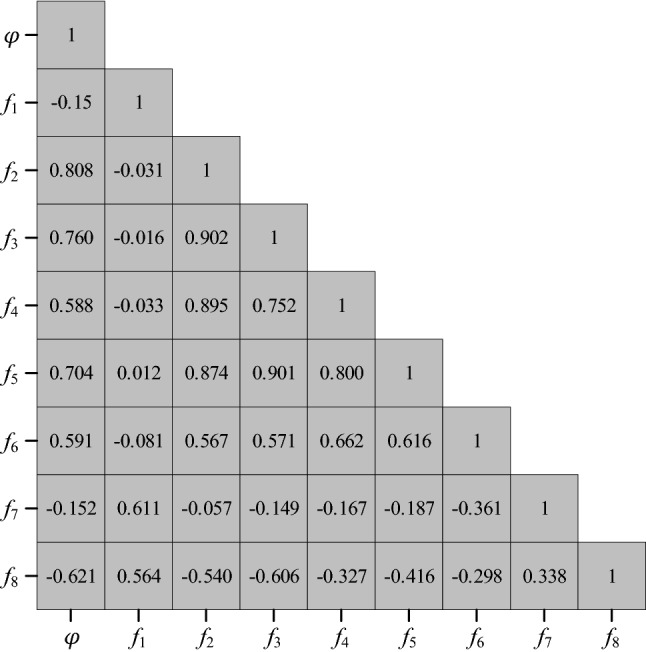
Correlation analysis matrix.

As shown in Fig. 10, the correlations between some independent variables are higher than those between the dependent variable and some independent variables. For example, the correlation between *f*_2_ and *φ* is 0.808, and the correlation between *f*_2_ and *f*_3_ is 0.902. To reduce multicollinearity, *PCR* is implemented.

As shown in Table 6, the eigenvalues of *C*_1_, *C*_2_, and *C*_3_ are all larger than 1, and their cumulative variance accounts for more than 92%. Therefore, three principal components (*C*_1_, *C*_2_, and *C*_3_) are selected to represent the 8 independent variables. Then the regression model is established according to Eq. (10). The detailed parameters of the regression equation are listed in Table 7. The *p* for the regression model is equal to 0, which means the regression model is significant. The *p* values for the coefficients of *C*_1_, *C*_2_, and *C*_3_ are all smaller than 0.01, which means that *C*_1_, *C*_2_, and *C*_3_ all have a significant effect on *φ* within a 99% confidence interval. The variance inflation factor (VIF) for the coefficients of *C*_1_, *C*_2_, and *C*_3_ are all equal to 1, which is smaller than 5. Therefore, the multicollinearity in this model is relatively weak.

**Table 6:** Cumulative variance of the principal components.

Component	Eigenvalue	Variance%	Cumulative variance %
*C*_1_	4.46	55.74	55.74
*C*_2_	1.79	22.38	78.12
*C*_3_	1.01	14.33	92.45
*C*_4_	0.36	3.44	95.89
*C*_5_	0.34	2.22	98.11
*C*_6_	0.16	0.95	99.06
*C*_7_	0.05	0.66	99.72
*C*_8_	0.02	0.28	100.000

**Table 7:** Detailed parameters of the regression model.

Parameter	Description	*t*	VIF	*p*	F	*R*^2^
model	*φ* = 0.803 *C*_1_ + 0.023 *C*_2_-0.0152 *C*_3_ + 0.063	–	–	0.000	14.132	0.806
*C*_1_	0.803	6.395	1	0.000	–	–
*C*_2_	0.023	4.710	1	0.003	–	–
*C*_3_	−0.152	−19.62	1	0.000	–	–

Then the coefficients for the eight kinds of (anti-) motifs can be obtained according to Eq. (11). The relationship between product structural robustness and the frequency of eight kinds of (anti-) motifs is

$$\varphi = - 0.{17}f_{{1}}^{^{\prime}} + 0.{78}f_{{2}}^{^{\prime}} + 0.{77}f_{{3}}^{^{\prime}} + 0.{73}f_{{4}}^{^{\prime}} + 0.{7}0f_{{5}}^{^{\prime}} + 0.{51}f_{{6}}^{^{\prime}} - 0.{18}f_{{7}}^{^{\prime}} - 0.{58}f_{{8}}^{^{\prime}} + 0.0{63}.$$

If the coefficient is larger than 0, then the corresponding (anti-) motif has a positive effect on product structural robustness; in contrast, if the coefficient is smaller than 0, the corresponding (anti-) motif is negative for product structural robustness. The larger the absolute value of the coefficient is, the more significant the corresponding (anti-) motif’s effect on product structural robustness. For example, the coefficients of *f*_1_^*'*^, *f*_7_^*'*^, and *f*_8_^*'*^ are smaller than 0, so the corresponding (anti-) motifs of *M*_1_, *M*_7_, and *M*_8_ are all negative on product structural robustness. Meanwhile, the absolute value of the coefficient for *f*_8_^*'*^ is 0.58, which is larger than that of *f*_1_^*'*^ and *f*_7_^*'*^. Therefore, the negative effect on the product structural robustness of *M*_8_ is much more significant. Similarly, the coefficients of *f*_2_^*'*^, *f*_3_^*'*^, *f*_4_^*'*^, *f*_5_^*'*^, and *f*_6_^*'*^ are all larger than 0. Therefore, the corresponding motifs of *M*_2_, *M*_3_, *M*_4_, *M*_5_, and *M*_6_ all play a positive role in product structural robustness. The absolute value of *f*_5_^*'*^ is the smallest in the coefficients of *M*_2_, *M*_3_, *M*_4_, *M*_5_, and *M*_6_. Thus, it has a relatively small positive influence on product structural robustness.

As shown in Table 8, four observations can be concluded. (1) All anti-motifs (*M*_1_ and *M*_7_) are negative for product structural robustness. (2) Most motifs have a positive effect on product structural robustness, except *M*_8_. (3) Motifs that contain a loop structure are positive for product structural robustness. (For example, motifs of *M*_2_, *M*_3_, *M*_4,_ and *M*_5_ have a three-node loop structure, and *M*_6_ has a four-node loop structure. They all have a positive effect on product structural robustness. In contrast, *M*_1_, *M*_7,_ and *M*_8_ do not have a loop structure and are negative for product structural robustness.) (4) The positive effect on product structural robustness of motifs with the three-node loop structure is higher than that of motifs with a four-node loop structure. Motifs (*M*_2_, *M*_3_, *M*_4_, and *M*_5_) with a three-node loop structure all have a coefficient larger than 0.7, and the motif (*M*_6_) with a four-node loop structure only has a coefficient of 0.51.

**Table 8:** Effect of (anti-) motifs on product structural robustness.

No	Coefficient	Effect	(Anti-) Motif	Type
*M*_2_	0.78	Positive	Motif	
*M*_3_	0.77		Motif	
*M*_4_	0.73		Motif	
*M*_5_	0.70		Motif	
*M*_6_	0.51		Motif	
*M*_8_	−0.58	Negative	Motif	
*M*_1_	−0.17		Anti-motif	
*M*_7_	−0.18		Anti-motif	

### Improve product structural robustness based on three motif-based strategies

According to the strategies proposed in “Motif-based strategies to improve product structural robustness” section and the observations concluded in “Robustness of product structural networks” section, three detailed motif-based strategies are analysed to improve product structural robustness.

#### Strategy 1: Increase the frequencies of motifs with loop structures in product structural networks

**Figure 11:**
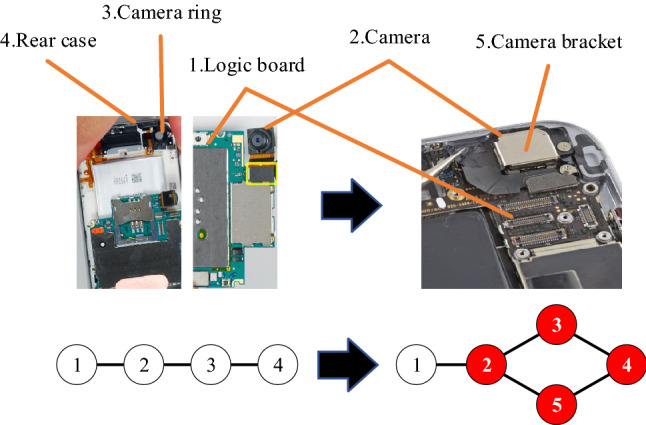
A four-node loop structure was added to keep the camera stable. (Adapted in part with free permission from iFixit, https://www.ifixit.com/Teardown/iPhone/. Licenced under the CC BY open access licence).

As shown in Fig. 11, the camera is connected to the logic board and protected by the camera ring at the original product generation. The camera ring can only limit the freedom of the camera in five directions, which leads to the possibility of longitudinal loosening of the camera. Then, the camera tends to be structurally unstable and it may not fully implement its functions. In later generations, designers added a camera bracket to secure the camera. The camera bracket forms a four-node loop structure with other components to better maintain the stability of the camera without affecting the structure or function of other components. Therefore, adding motifs with loop structures is beneficial for the improvement of product structural robustness.

**Figure 12:**
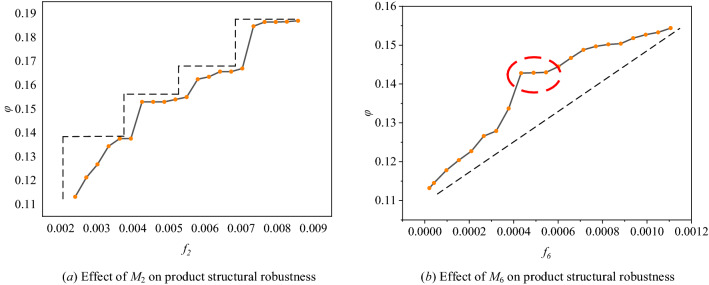
Effect of motifs with the three-node and four-node loop structure on *φ.*

Meanwhile, the simulation of increasing the frequencies of motifs with the loop structure is implemented to analyse its effect on product structural robustness. As shown in Fig. 12, we increase the frequency of motifs with three-node and four-node loop structures in the structural network of P1. (Remarkably, because the correlation coefficients between *M*_2_, *M*_3_, *M*_4_, and *M*_5_ are high (see Fig. 10); the coefficients of the regression model for them are very close (see Table 8); and they are all formed by a three-node loop structure, *M*_2_ is selected to represent *M*_3_, *M*_4_, and *M*_5_ in this section). As shown in Fig. 12a, as the frequency of *M*_2_ gradually increases, product structural robustness also increases followed by a step pattern. This step pattern is caused by the different effects on the network connectivity of motifs that are formed by nodes with different properties.

**Figure 13:**
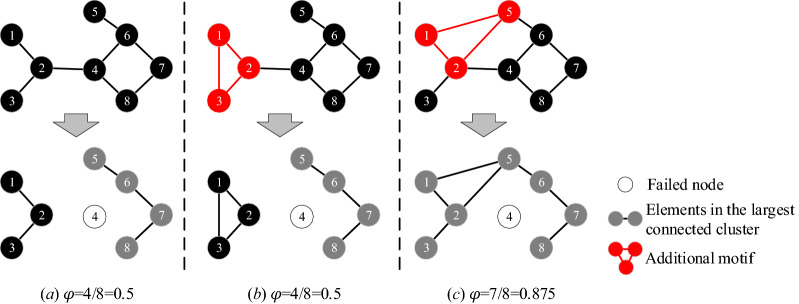
Robustness of the network under different formations of *M*_2_.

As shown in Fig. 13b, the added motif formed by node 2 and its hanging nodes 1 and 3 does not effectively enhance the connectivity of the network. (Hanging nodes are those nodes that only have one edge). Therefore, the largest connected cluster does not significantly increase when the network suffers from *FKC*, which results in the improvement of robustness being not obvious compared with the original network (see Fig. 13a). As shown in Fig. 13c, if *M*_2_ is formed by handing node 1 and other nodes (not the hanging nodes of node 2), the connectivity of the network is significantly improved. Then, the network robustness is improved too. As shown in Fig. 7, there are many hanging nodes in the product structural network. Therefore, when the added *M*_2_ is formed by the node and its two hanging nodes, product structural robustness only increases slightly, as shown in Fig. 12a.

Similarly, as shown in Fig. 12b, with the increase in the frequency of the four-node loop structure (*M*_6_), product structural robustness also increases. Although there still exists a step pattern in the uptrend, the main trend is a rising line. This is because the four-node loop structure makes it easier for those hanging nodes to connect with other nodes. Therefore, it is easier to improve the overall connectivity and the robustness of the network.

#### Strategy 2: Reduce the frequencies of (anti-) motifs of ***M***_1_, ***M***_7_, and ***M***_8_ in product structural networks

**Figure 14:**
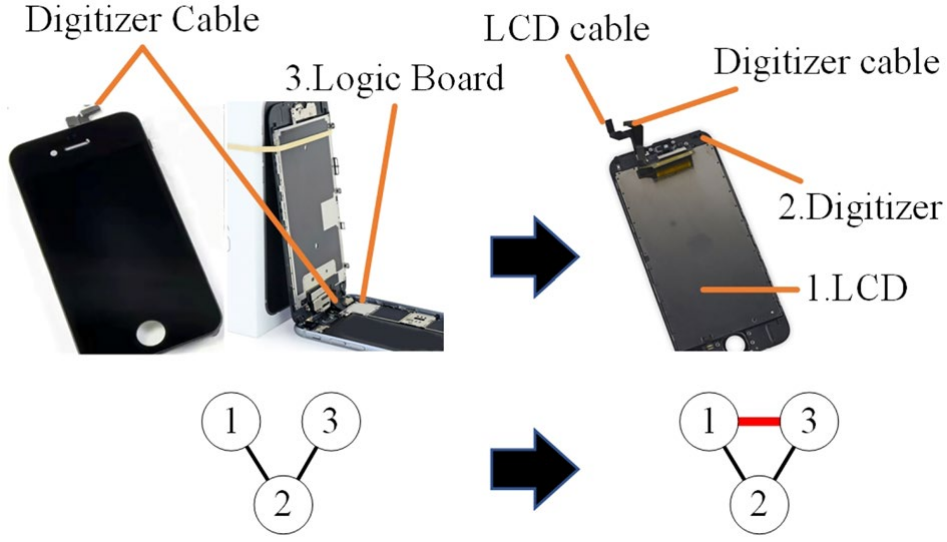
Reduce the frequency of *M*_1_ to improve product structural robustness. (Adapted in part with free permission from iFixit, https://www.ifixit.com/Teardown/iPhone/. Licenced under the CC BY open access licence).

Both instance analysis and simulation are implemented to analyse the impact of this strategy on improving product structural robustness. As shown in Fig. 14, in the left product, the LCD connects to the digitizer and they both transmit the information to the logic board through the digitizer cable. The LCD, the digitizer (with digitizer cable), and the logic board form an anti-motif of *M*_1_. If the digitizer fails, the LCD cannot work either. In contrast, as shown in the product in the right part of Fig. 14, the LCD transmits the information to the logic board by the LCD cable. The structure of *M*_1_ changes to *M*_2_ and the frequency of *M*_1_ is reduced. Then, the LCD and the digitizer can work independently and the failure of one of them does not affect the work of the other. Therefore, product structural robustness is improved by reducing the frequency of *M*_2_. Similarly, the frequency of *M*_7_ and *M*_8_ can also be reduced by changing their structure to improve product structural robustness.

**Figure 15:**
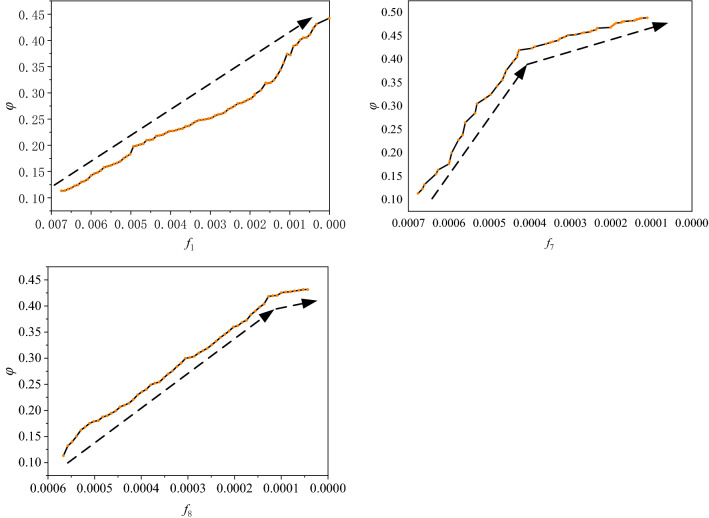
Change of *φ* with the reduction of *f*_1_, *f*_7_, and *f*_8_.

As shown in Fig. 15, with the reduction of the frequencies of these (anti-) motifs, product structural robustness gradually increases. For example, with the reduction of the frequency of *M*_1_, product structural robustness is linearly increasing. Product structural robustness sharply increases when the frequency of *M*_7_ decreases from 0.007 to 0.004; thereafter, the growth rate becomes lower. This is because the entire network connectivity increases quickly with the change in the frequency of *M*_7_; then, when the frequency of *M*_7_ reduces to a certain degree, the network connectivity increases slowly. Similarly, as the frequency of *M*_8_ gradually decreases, product structural robustness increases rapidly first and then relatively slowly.

#### Strategy 3: Protecting the key components and fragile components in (anti-) motifs

As shown in Table 9, M1 consists of a key component (rear case) and two other components (lightning connector and loudspeaker). The rear case is important in fixing various components. If it fails, both the lightning connector and loudspeaker cannot remain stable. Compared with *M*_1_, *M*_8_ also consists of a key component and many other components with no relationship. As shown in Table 9, many chips are connected to the logic board to accomplish various functions. If the logic board fails, all the chips will be disabled, and the smartphone cannot be used. The stability of *M*_8_ has a significant influence on product function performance. The structure of *M*_7_ is a single link structure to perform a specific function. For example, the function flow in Table 9 about *M*_7_ can be represented as follows: battery → logic board → lighting connector cable → lightning connector. If any component in the function flow fails (especially the lighting connector cable which is fragile), this function cannot be performed. To keep the product much more robust, designers should focus on the key components and fragile components in these (anti-) motifs. Meanwhile, designers need to develop a specific plan for the protection and regular inspections.

**Table 9:**
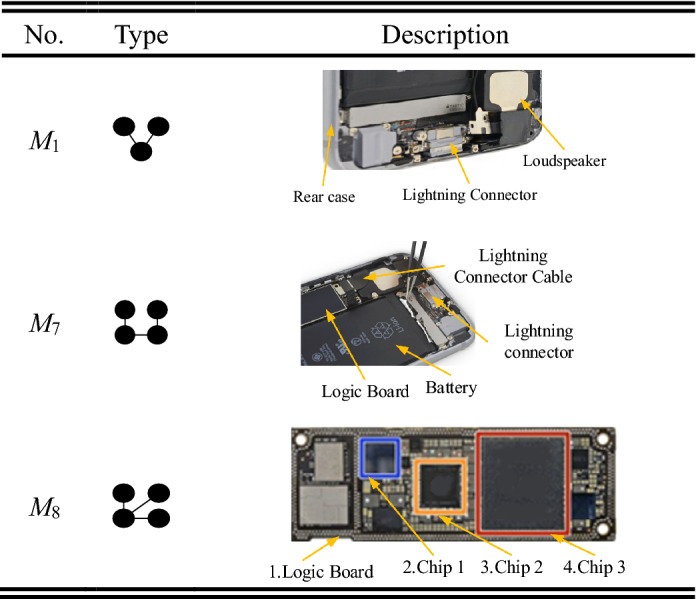
Examples of eight kinds of (anti-) motifs (Adapted in part with free permission from iFixit, https://www.ifixit.com/Teardown/iPhone/. Licenced under the CC BY open access licence).

## Discussion

**Figure 16:**
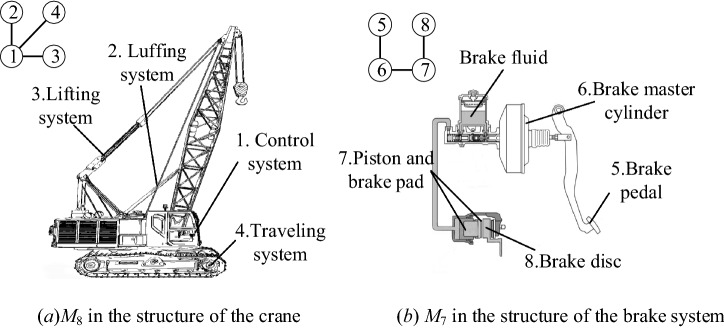
Motifs in common mechanical systems.

Improving product structural robustness is essential for the entire product lifetime. Compared with traditional methods, motifs provide a novel perspective to analyse product structural robustness. Any product can be regarded as a networked system, and each product structural network is formed by many different motifs. These motifs widely exist in the product structure and directly influence product structural characteristics. Therefore, applying motif-based strategies to improve product structural robustness is convenient and useful. For example, as shown in Fig. 16a, in complex mechanical products (such as cranes), the control system is connected with many other components to perform various complex functions. If the control system fails, the functions of luffing, lifting, and rotating of the crane may not work. Based on strategy 3, the control systems should be protected and checked regularly. Similarly, as shown in Fig. 16b, the brake system can be simplified as a structure of *M*_7_ (brake pedal → brake master cylinder → piston and brake pad → brake disc). Wherein, the brake pad has a high degree of wear and tear. According to strategy 3, brake pads need to be thickened, checked, and replaced regularly to improve product structural robustness.

Although motif-based strategies are useful to improve product structural robustness, some deficiencies of the method in this research should be claimed and discussed. First, there are possibly unidirectional flows between nodes, which are not fully analysed in this study. This issue may result in the research results not completely revealing the relationship between motifs and product structural robustness. A better measure is to establish a directed network model when the functional flows in the product can be easily acquired. In addition, the simulation for the increase or decrease of (anti-) motifs is based on the characteristics of product structural networks. It does not completely consider the actual functional and structural relationship between components. Some added (anti-) motifs may not truly reflect the product properties. Therefore, when applying motif-based strategies to improve product structural robustness, the influence of the change in (anti-) motifs on the actual function and structure of the product should be fully considered.

## Conclusion and future work

Product structural networks are vulnerable to *FKC*. To improve product structural robustness, a method based on network motifs is proposed in this study, which is concluded as follows.

1. Due to the lack of universal and typical structure optimization strategies for improving product structural robustness, this study proposed a product structural robustness analysis method based on motifs. Through this method, the effect of motifs on product structural robustness is uncovered. The results then provide designers with specific and useful strategies to improve product structural robustness.
2. In the proposed methodology, *PCR* is applied to reduce the potential multicollinearity among different motifs and then investigate the correlations between motifs and product structural robustness. In this process, the frequency of motifs is defined, which eliminates the influence of network size.
3. Taking the robustness analysis of 25 generations of smartphones as an example, the validity and feasibility of the proposed method are verified. Three strategies for the improvement of product structural robustness are deduced according to the result analysis.

Nevertheless, with the increasing or decreasing of typical motifs in product structure, although the robustness could be improved, the complexity of product structure would also be increased. Then, the cost of product research and development will be increased. Thus, in future work, the motif-based method to simultaneously improve product structural robustness and complexity will be further studied.

## Supplementary Information

Supplementary Information.

## Data Availability

All data generated or analysed during this study are included in this published article (and its Supplementary Information files).
