# Supplementary material for: Improve the product structural robustness based on network motifs in product development
Source: Sci Rep. 2022 Jun 28;12:10916. doi: 10.1038/s41598-022-15056-2 (PMC9240074; doi:10.1038/s41598-022-15056-2)
Supplement: Supplementary file 1 — Supplementary Information. [file 41598_2022_15056_MOESM1_ESM.pdf]

The Supplementary Information files provide the detailed information of the relationship between components for each product. If component 1 and component 2 have a relationship, then it is remarked as 1 2 1. The first number 1 represents component 1, the second number 2 represents component 2, and the last number 1 means there is a relationship between component 1 and component 2

Relationship between components in product 1

1	2	1
1	6	1
1	20	1
2	3	1
2	5	1
2	6	1
2	7	1
2	17	1
2	20	1
3	4	1
3	6	1
4	20	1
5	6	1
5	10	1
5	11	1
5	16	1
6	9	1
6	10	1
6	12	1
6	14	1
6	15	1
6	16	1
6	17	1
6	18	1
6	20	1
6	21	1
6	44	1
6	45	1
7	8	1
8	17	1
8	19	1
9	10	1
9	21	1
10	11	1
10	13	1
10	35	1
10	36	1
10	37	1
10	38	1
10	39	1
10	40	1
10	41	1
10	42	1
10	46	1
10	47	1
11	16	1
11	18	1
11	43	1
12	13	1
12	20	1
14	17	1
14	19	1
15	17	1
15	19	1
16	17	1
16	18	1
16	19	1
17	18	1
17	19	1
18	43	1
19	20	1
20	21	1
20	22	1
20	24	1
20	30	1
20	31	1
20	34	1
21	23	1
21	25	1
21	26	1
21	27	1
21	28	1
21	29	1
21	32	1
21	33	1
21	44	1
21	45	1
21	47	1
34	35	1
36	37	1
36	47	1
38	39	1
39	47	1
40	41	1
41	47	1
42	46	1
42	47	1
43	44	1

Relationship between components in product 2

1	2	1
1	4	1
1	9	1
1	18	1
2	3	1
2	4	1
2	6	1
2	7	1
2	8	1
2	10	1
2	18	1
3	4	1
3	5	1
4	5	1
5	18	1
6	10	1
6	16	1
6	34	1
6	35	1
6	36	1
6	37	1
6	38	1
6	39	1
6	40	1
7	8	1
8	17	1
9	10	1
9	18	1
10	12	1
10	15	1
10	16	1
10	18	1
10	33	1
10	34	1
10	35	1
10	36	1
10	37	1
10	38	1
10	39	1
10	40	1
10	41	1
10	42	1
10	43	1
10	44	1
11	12	1
11	18	1
13	16	1
13	17	1
14	16	1
14	17	1
15	16	1
15	17	1
16	17	1
16	41	1
17	18	1
18	19	1
18	20	1
18	21	1
18	22	1
18	23	1
18	24	1
18	25	1
18	26	1
18	27	1
18	28	1
18	29	1
18	30	1
18	31	1
18	32	1
18	41	1
18	42	1
18	43	1
18	44	1
32	33	1
34	35	1
34	44	1
36	37	1
37	44	1
38	39	1
39	44	1
40	44	1

Relationship between components in product 3

1	2	1
1	4	1
1	9	1
1	18	1
2	3	1
2	4	1
2	6	1
2	7	1
2	8	1
2	10	1
2	18	1
3	4	1
3	5	1
4	5	1
5	18	1
6	10	1
6	16	1
6	34	1
6	35	1
6	36	1
6	37	1
6	38	1
6	39	1
6	40	1
7	8	1
8	17	1
9	10	1
9	18	1
10	12	1
10	15	1
10	16	1
10	18	1
10	33	1
10	34	1
10	35	1
10	36	1
10	37	1
10	38	1
10	39	1
10	40	1
10	41	1
10	42	1
10	43	1
10	44	1
11	12	1
11	18	1
13	16	1
13	17	1
14	16	1
14	17	1
15	16	1
15	17	1
16	17	1
16	41	1
17	18	1
18	19	1
18	20	1
18	21	1
18	22	1
18	23	1
18	24	1
18	25	1
18	26	1
18	27	1
18	28	1
18	29	1
18	30	1
18	31	1
18	32	1
18	41	1
18	42	1
18	43	1
18	44	1
32	33	1
34	35	1
34	44	1
36	37	1
37	44	1
38	39	1
39	44	1
40	44	1

Relationship between components in product 4

1	2	1
1	4	1
1	23	1
1	52	1
2	4	1
2	5	1
2	10	1
2	23	1
2	52	1
3	4	1
3	23	1
4	7	1
4	8	1
4	9	1
4	10	1
4	11	1
4	12	1
4	14	1
4	15	1
4	16	1
4	18	1
4	19	1
4	20	1
4	23	1
4	39	1
4	40	1
4	41	1
4	43	1
4	44	1
4	45	1
4	46	1
4	47	1
4	52	1
4	53	1
4	55	1
5	6	1
6	21	1
7	8	1
7	23	1
7	51	1
8	9	1
8	23	1
9	13	1
9	14	1
10	11	1
10	23	1
12	13	1
12	14	1
12	23	1
15	16	1
15	20	1
15	21	1
15	48	1
15	49	1
17	18	1
17	20	1
17	21	1
19	20	1
19	21	1
20	48	1
21	22	1
21	23	1
22	23	1
23	24	1
23	25	1
23	26	1
23	27	1
23	28	1
23	29	1
23	30	1
23	31	1
23	32	1
23	33	1
23	34	1
23	35	1
23	36	1
23	37	1
23	38	1
23	42	1
23	49	1
23	50	1
23	51	1
23	52	1
23	54	1
38	39	1
40	41	1
40	42	1
42	52	1
42	55	1
43	44	1
44	54	1
45	46	1
46	54	1
47	53	1
47	54	1
48	49	1
49	50	1
52	54	1
53	54	1

Relationship between components in product 5

1	2	1
1	4	1
1	23	1
1	56	1
2	4	1
2	5	1
2	10	1
2	23	1
2	56	1
3	4	1
3	23	1
4	7	1
4	8	1
4	9	1
4	10	1
4	11	1
4	12	1
4	14	1
4	15	1
4	16	1
4	18	1
4	19	1
4	20	1
4	23	1
4	39	1
4	40	1
4	41	1
4	43	1
4	44	1
4	45	1
4	46	1
4	47	1
4	52	1
4	53	1
4	55	1
5	6	1
6	21	1
7	8	1
7	23	1
7	51	1
8	9	1
8	23	1
9	13	1
9	14	1
10	11	1
10	23	1
12	13	1
12	14	1
12	23	1
15	16	1
15	20	1
15	21	1
15	48	1
15	49	1
17	18	1
17	20	1
17	21	1
19	20	1
19	21	1
20	48	1
21	22	1
21	23	1
22	23	1
23	24	1
23	25	1
23	26	1
23	27	1
23	28	1
23	29	1
23	30	1
23	31	1
23	32	1
23	33	1
23	34	1
23	35	1
23	36	1
23	37	1
23	38	1
23	42	1
23	48	1
23	49	1
23	50	1
23	51	1
23	53	1
23	54	1
23	56	1
38	39	1
40	41	1
40	42	1
43	44	1
44	54	1
45	46	1
46	54	1
47	54	1
48	49	1
49	50	1
54	55	1
54	56	1

Relationship between components in product 6

1	2	1
1	4	1
1	23	1
1	56	1
2	4	1
2	5	1
2	10	1
2	23	1
2	56	1
3	4	1
3	23	1
4	7	1
4	8	1
4	9	1
4	10	1
4	11	1
4	12	1
4	14	1
4	15	1
4	16	1
4	18	1
4	19	1
4	20	1
4	23	1
4	39	1
4	40	1
4	41	1
4	43	1
4	44	1
4	45	1
4	46	1
4	47	1
4	52	1
4	53	1
4	55	1
5	6	1
6	21	1
7	8	1
7	23	1
7	50	1
7	51	1
8	9	1
8	23	1
9	13	1
9	14	1
10	11	1
10	23	1
12	13	1
12	14	1
12	23	1
15	16	1
15	20	1
15	21	1
15	48	1
15	49	1
17	18	1
17	20	1
17	21	1
19	20	1
19	21	1
20	48	1
21	22	1
21	23	1
22	23	1
23	24	1
23	25	1
23	26	1
23	27	1
23	28	1
23	29	1
23	30	1
23	31	1
23	32	1
23	33	1
23	34	1
23	35	1
23	36	1
23	37	1
23	38	1
23	42	1
23	49	1
23	50	1
23	51	1
23	53	1
23	54	1
23	56	1
38	39	1
40	41	1
40	42	1
43	44	1
44	54	1
45	46	1
46	54	1
47	52	1
47	54	1
48	49	1
49	50	1
54	55	1
54	56	1

Relationship between components in product 7

1	2	1
1	3	1
1	12	1
1	25	1
2	3	1
2	4	1
2	5	1
2	7	1
2	10	1
2	11	1
2	12	1
2	13	1
3	13	1
3	25	1
4	12	1
4	13	1
5	6	1
5	7	1
6	7	1
6	23	1
8	9	1
8	25	1
8	52	1
9	10	1
10	16	1
10	17	1
10	18	1
10	20	1
10	21	1
10	23	1
10	25	1
10	26	1
10	42	1
10	43	1
10	44	1
10	45	1
10	46	1
10	47	1
10	48	1
10	49	1
10	50	1
10	53	1
10	55	1
10	56	1
11	12	1
11	13	1
13	25	1
14	15	1
14	16	1
14	17	1
14	25	1
15	17	1
15	25	1
17	25	1
18	21	1
18	22	1
18	23	1
18	51	1
18	52	1
19	20	1
19	22	1
19	23	1
21	22	1
21	23	1
22	23	1
22	50	1
23	24	1
23	25	1
23	50	1
24	25	1
25	26	1
25	27	1
25	28	1
25	29	1
25	30	1
25	31	1
25	32	1
25	33	1
25	34	1
25	35	1
25	36	1
25	37	1
25	38	1
25	39	1
25	40	1
25	41	1
25	52	1
25	54	1
25	56	1
25	57	1
25	58	1
41	42	1
44	45	1
44	56	1
46	47	1
47	56	1
48	49	1
49	56	1
51	52	1
51	56	1
53	54	1
55	56	1

Relationship between components in product 8

1	2	1
1	3	1
1	12	1
1	13	1
1	24	1
2	3	1
2	4	1
2	5	1
2	7	1
2	10	1
2	11	1
2	12	1
2	13	1
3	13	1
3	24	1
4	12	1
4	13	1
5	6	1
5	7	1
6	7	1
6	23	1
8	9	1
8	23	1
8	51	1
9	10	1
9	24	1
10	15	1
10	16	1
10	17	1
10	18	1
10	20	1
10	21	1
10	23	1
10	24	1
10	25	1
10	41	1
10	42	1
10	43	1
10	44	1
10	45	1
10	46	1
10	47	1
10	48	1
10	49	1
10	52	1
10	54	1
10	55	1
11	12	1
11	13	1
13	24	1
14	15	1
14	16	1
14	24	1
17	55	1
18	21	1
18	22	1
18	23	1
18	50	1
18	51	1
19	20	1
19	22	1
19	23	1
21	22	1
21	23	1
22	23	1
22	49	1
23	24	1
23	49	1
24	25	1
24	26	1
24	27	1
24	28	1
24	29	1
24	30	1
24	31	1
24	32	1
24	33	1
24	34	1
24	35	1
24	36	1
24	37	1
24	38	1
24	39	1
24	40	1
24	51	1
24	53	1
24	55	1
25	56	1
25	57	1
40	41	1
43	44	1
43	55	1
45	46	1
46	55	1
47	48	1
48	55	1
50	51	1
50	55	1
52	53	1
54	55	1

Relationship between components in product 9

1	2	1
1	3	1
1	14	1
1	15	1
1	26	1
2	3	1
2	4	1
2	5	1
2	7	1
2	12	1
2	13	1
2	14	1
2	15	1
3	15	1
3	26	1
4	14	1
4	15	1
5	6	1
5	7	1
5	8	1
6	7	1
6	9	1
6	25	1
10	11	1
10	25	1
10	26	1
10	55	1
11	12	1
11	26	1
12	16	1
12	17	1
12	18	1
12	19	1
12	20	1
12	22	1
12	25	1
12	26	1
12	45	1
12	46	1
12	47	1
12	48	1
12	49	1
12	50	1
12	51	1
12	52	1
12	53	1
12	56	1
12	58	1
12	59	1
13	14	1
13	15	1
15	26	1
16	17	1
16	18	1
16	26	1
19	59	1
20	23	1
20	24	1
20	25	1
20	54	1
20	55	1
21	22	1
21	24	1
21	25	1
23	24	1
23	25	1
24	25	1
24	53	1
25	26	1
25	53	1
26	27	1
26	28	1
26	29	1
26	30	1
26	31	1
26	32	1
26	33	1
26	34	1
26	35	1
26	36	1
26	37	1
26	38	1
26	39	1
26	40	1
26	41	1
26	42	1
26	43	1
26	44	1
26	55	1
26	57	1
26	59	1
44	45	1
47	48	1
47	59	1
49	50	1
50	59	1
51	52	1
52	59	1
54	55	1
54	59	1
56	57	1
58	59	1

Relationship between components in product 10

1	2	1
1	3	1
1	6	1
1	26	1
2	3	1
2	4	1
2	5	1
2	7	1
2	11	1
2	12	1
2	13	1
2	14	1
3	6	1
3	14	1
3	26	1
4	13	1
4	14	1
5	6	1
5	7	1
5	8	1
6	7	1
6	26	1
9	10	1
9	25	1
9	26	1
9	59	1
10	11	1
10	26	1
11	15	1
11	16	1
11	17	1
11	18	1
11	20	1
11	22	1
11	23	1
11	25	1
11	26	1
11	47	1
11	48	1
11	49	1
11	50	1
11	51	1
11	52	1
11	53	1
11	54	1
11	55	1
11	56	1
11	57	1
11	58	1
11	62	1
11	63	1
11	64	1
12	13	1
12	14	1
14	26	1
15	16	1
15	17	1
15	26	1
18	19	1
18	63	1
19	64	1
20	24	1
20	25	1
20	58	1
20	59	1
21	22	1
21	24	1
21	25	1
23	24	1
23	25	1
24	25	1
24	57	1
25	26	1
25	57	1
25	58	1
25	62	1
26	27	1
26	28	1
26	29	1
26	30	1
26	31	1
26	32	1
26	33	1
26	34	1
26	35	1
26	36	1
26	37	1
26	38	1
26	39	1
26	40	1
26	41	1
26	42	1
26	43	1
26	44	1
26	45	1
26	46	1
26	56	1
26	59	1
26	60	1
26	61	1
26	63	1
46	47	1
49	50	1
49	51	1
49	63	1
50	51	1
52	53	1
53	56	1
54	55	1
55	56	1
58	59	1
60	61	1
63	64	1

Relationship between components in product 11

1	2	1
1	3	1
1	6	1
1	27	1
2	3	1
2	4	1
2	5	1
2	7	1
2	11	1
2	12	1
2	13	1
2	14	1
3	6	1
3	14	1
3	27	1
4	13	1
4	14	1
5	6	1
5	7	1
5	8	1
6	7	1
6	27	1
9	10	1
9	25	1
9	27	1
9	62	1
10	11	1
10	27	1
11	15	1
11	16	1
11	17	1
11	18	1
11	20	1
11	22	1
11	23	1
11	25	1
11	27	1
11	50	1
11	51	1
11	52	1
11	53	1
11	54	1
11	55	1
11	56	1
11	57	1
11	58	1
11	59	1
11	60	1
11	61	1
11	65	1
11	66	1
11	67	1
12	13	1
12	14	1
14	27	1
15	16	1
15	17	1
15	27	1
18	19	1
18	66	1
19	67	1
20	24	1
20	25	1
20	61	1
20	62	1
21	22	1
21	24	1
21	25	1
23	24	1
23	25	1
24	25	1
24	60	1
25	26	1
25	27	1
25	60	1
25	61	1
25	65	1
26	27	1
27	28	1
27	29	1
27	30	1
27	31	1
27	32	1
27	33	1
27	34	1
27	35	1
27	36	1
27	37	1
27	38	1
27	39	1
27	40	1
27	41	1
27	42	1
27	43	1
27	44	1
27	45	1
27	46	1
27	47	1
27	48	1
27	49	1
27	59	1
27	62	1
27	63	1
27	64	1
27	66	1
49	50	1
52	53	1
52	54	1
52	66	1
53	54	1
55	56	1
56	59	1
57	58	1
58	59	1
61	62	1
63	64	1
66	67	1

Relationship between components in product 12

1	2	1
1	3	1
1	6	1
1	27	1
2	3	1
2	4	1
2	5	1
2	7	1
2	11	1
2	12	1
2	13	1
2	14	1
3	6	1
3	14	1
3	27	1
4	13	1
4	14	1
5	6	1
5	7	1
5	8	1
6	7	1
6	27	1
9	10	1
9	26	1
9	27	1
9	59	1
10	11	1
10	27	1
11	15	1
11	16	1
11	17	1
11	18	1
11	20	1
11	23	1
11	24	1
11	26	1
11	27	1
11	46	1
11	47	1
11	48	1
11	49	1
11	50	1
11	51	1
11	52	1
11	53	1
11	54	1
11	55	1
11	57	1
11	62	1
11	63	1
11	64	1
12	13	1
12	14	1
14	27	1
15	16	1
15	17	1
15	27	1
18	19	1
18	63	1
19	64	1
20	25	1
20	26	1
20	57	1
20	59	1
21	23	1
21	25	1
21	26	1
22	23	1
22	25	1
22	26	1
24	25	1
24	26	1
25	26	1
25	55	1
26	27	1
26	55	1
26	57	1
26	62	1
27	28	1
27	29	1
27	30	1
27	31	1
27	32	1
27	33	1
27	34	1
27	35	1
27	36	1
27	37	1
27	38	1
27	39	1
27	40	1
27	41	1
27	42	1
27	43	1
27	44	1
27	45	1
27	56	1
27	58	1
27	59	1
27	60	1
27	61	1
27	63	1
45	46	1
48	49	1
48	50	1
48	63	1
49	50	1
51	52	1
52	63	1
53	54	1
54	63	1
57	59	1
58	60	1
60	61	1
63	64	1

Relationship between components in product 13

1	2	1
1	3	1
1	6	1
1	27	1
2	3	1
2	4	1
2	5	1
2	7	1
2	11	1
2	12	1
2	13	1
2	14	1
3	6	1
3	14	1
3	27	1
4	13	1
4	14	1
5	6	1
5	7	1
5	8	1
6	7	1
6	27	1
9	10	1
9	26	1
9	27	1
9	59	1
10	11	1
10	27	1
11	15	1
11	16	1
11	17	1
11	18	1
11	20	1
11	23	1
11	24	1
11	26	1
11	27	1
11	46	1
11	47	1
11	48	1
11	49	1
11	50	1
11	51	1
11	52	1
11	53	1
11	54	1
11	55	1
11	57	1
11	62	1
11	63	1
11	64	1
12	13	1
12	14	1
14	27	1
15	16	1
15	17	1
15	27	1
18	19	1
18	63	1
19	64	1
20	25	1
20	26	1
20	57	1
20	59	1
21	23	1
21	25	1
21	26	1
22	23	1
22	25	1
22	26	1
24	25	1
24	26	1
25	26	1
25	55	1
26	27	1
26	55	1
26	57	1
26	62	1
27	28	1
27	29	1
27	30	1
27	31	1
27	32	1
27	33	1
27	34	1
27	35	1
27	36	1
27	37	1
27	38	1
27	39	1
27	40	1
27	41	1
27	42	1
27	43	1
27	44	1
27	45	1
27	56	1
27	58	1
27	59	1
27	60	1
27	61	1
27	63	1
45	46	1
48	49	1
48	50	1
48	63	1
49	50	1
51	52	1
52	63	1
53	54	1
54	63	1
57	59	1
58	60	1
60	61	1
63	64	1

Relationship between components in product 14

1	2	1
1	3	1
1	14	1
1	15	1
1	26	1
2	3	1
2	4	1
2	5	1
2	7	1
2	12	1
2	13	1
2	14	1
2	15	1
2	26	1
3	15	1
3	26	1
4	14	1
4	15	1
5	6	1
5	7	1
5	8	1
6	7	1
6	9	1
6	25	1
10	11	1
10	25	1
10	26	1
10	55	1
11	12	1
11	26	1
12	16	1
12	17	1
12	18	1
12	19	1
12	20	1
12	22	1
12	23	1
12	25	1
12	26	1
12	45	1
12	46	1
12	47	1
12	48	1
12	49	1
12	50	1
12	51	1
12	52	1
12	53	1
12	56	1
12	58	1
12	59	1
13	14	1
13	15	1
15	26	1
16	17	1
16	18	1
16	26	1
19	59	1
20	23	1
20	24	1
20	25	1
20	54	1
20	55	1
21	22	1
21	24	1
21	25	1
23	24	1
23	25	1
24	25	1
24	53	1
25	26	1
25	53	1
26	27	1
26	28	1
26	29	1
26	30	1
26	31	1
26	32	1
26	33	1
26	34	1
26	35	1
26	36	1
26	37	1
26	38	1
26	39	1
26	40	1
26	41	1
26	42	1
26	43	1
26	44	1
26	55	1
26	57	1
26	59	1
44	45	1
47	48	1
47	59	1
49	50	1
50	59	1
51	52	1
52	59	1
54	55	1
56	57	1
58	59	1

Relationship between components in product 15

1	2	1
1	8	1
1	12	1
1	28	1
2	3	1
2	4	1
2	5	1
2	6	1
2	8	1
2	10	1
2	11	1
2	12	1
2	13	1
2	28	1
3	12	1
3	13	1
4	5	1
4	6	1
4	7	1
5	6	1
8	9	1
8	27	1
8	28	1
8	60	1
8	61	1
9	10	1
9	28	1
10	15	1
10	16	1
10	17	1
10	18	1
10	19	1
10	20	1
10	23	1
10	24	1
10	25	1
10	27	1
10	28	1
10	50	1
10	51	1
10	52	1
10	53	1
10	54	1
10	55	1
10	56	1
10	57	1
10	62	1
10	63	1
10	65	1
10	66	1
10	67	1
10	68	1
11	12	1
11	13	1
13	14	1
13	28	1
14	15	1
14	28	1
15	16	1
15	17	1
15	28	1
18	19	1
18	67	1
19	58	1
19	68	1
20	26	1
20	27	1
20	60	1
20	61	1
21	23	1
21	26	1
21	27	1
22	23	1
22	26	1
22	27	1
24	25	1
24	26	1
24	27	1
26	27	1
27	28	1
27	59	1
27	65	1
28	29	1
28	30	1
28	31	1
28	32	1
28	33	1
28	34	1
28	35	1
28	36	1
28	37	1
28	38	1
28	39	1
28	40	1
28	41	1
28	42	1
28	43	1
28	44	1
28	45	1
28	46	1
28	47	1
28	48	1
28	49	1
28	58	1
28	60	1
28	61	1
28	62	1
28	63	1
28	64	1
28	67	1
49	50	1
52	53	1
52	67	1
54	55	1
55	67	1
56	57	1
57	67	1
59	61	1
64	67	1
65	66	1
67	68	1

Relationship between components in product 16

1	2	1
1	8	1
1	12	1
1	29	1
2	3	1
2	4	1
2	5	1
2	6	1
2	8	1
2	10	1
2	11	1
2	12	1
2	13	1
2	29	1
3	12	1
3	13	1
4	5	1
4	6	1
4	7	1
5	6	1
8	9	1
8	28	1
8	29	1
8	66	1
8	67	1
9	10	1
9	29	1
10	15	1
10	16	1
10	17	1
10	18	1
10	19	1
10	20	1
10	21	1
10	24	1
10	25	1
10	26	1
10	28	1
10	29	1
10	55	1
10	56	1
10	57	1
10	58	1
10	59	1
10	60	1
10	61	1
10	62	1
10	63	1
10	64	1
10	68	1
10	70	1
10	71	1
10	72	1
10	73	1
10	74	1
10	75	1
11	12	1
11	13	1
13	14	1
13	29	1
14	15	1
14	16	1
14	29	1
15	17	1
15	18	1
15	29	1
16	17	1
16	18	1
16	29	1
19	20	1
19	72	1
20	64	1
20	73	1
21	27	1
21	28	1
21	66	1
21	67	1
22	24	1
22	27	1
22	28	1
23	24	1
23	27	1
23	28	1
25	26	1
25	27	1
25	28	1
27	28	1
28	29	1
28	65	1
28	70	1
29	30	1
29	31	1
29	32	1
29	33	1
29	34	1
29	35	1
29	36	1
29	37	1
29	38	1
29	39	1
29	40	1
29	41	1
29	42	1
29	43	1
29	44	1
29	45	1
29	46	1
29	47	1
29	48	1
29	49	1
29	50	1
29	51	1
29	52	1
29	53	1
29	54	1
29	64	1
29	66	1
29	67	1
29	68	1
29	69	1
29	72	1
29	74	1
29	75	1
54	55	1
55	57	1
58	59	1
58	72	1
60	61	1
61	72	1
62	63	1
63	72	1
65	67	1
69	72	1
70	71	1
72	73	1

Relationship between components in product 17

1	2	1
1	8	1
1	12	1
1	28	1
2	3	1
2	4	1
2	5	1
2	6	1
2	8	1
2	10	1
2	11	1
2	12	1
2	13	1
2	28	1
2	68	1
3	12	1
3	13	1
4	5	1
4	6	1
4	7	1
5	6	1
8	9	1
8	27	1
8	28	1
8	57	1
9	10	1
9	28	1
10	15	1
10	16	1
10	17	1
10	18	1
10	19	1
10	20	1
10	23	1
10	24	1
10	25	1
10	27	1
10	28	1
10	47	1
10	48	1
10	49	1
10	50	1
10	51	1
10	52	1
10	53	1
10	54	1
10	55	1
10	56	1
10	58	1
10	60	1
10	61	1
10	62	1
10	63	1
10	64	1
10	65	1
10	66	1
10	67	1
10	69	1
11	12	1
11	13	1
13	14	1
13	28	1
14	15	1
14	28	1
15	16	1
15	17	1
15	28	1
18	19	1
18	63	1
19	56	1
19	64	1
20	26	1
20	27	1
20	57	1
21	23	1
21	26	1
21	27	1
22	23	1
22	26	1
22	27	1
24	25	1
24	26	1
24	27	1
26	27	1
26	61	1
27	28	1
27	60	1
28	29	1
28	30	1
28	31	1
28	32	1
28	33	1
28	34	1
28	35	1
28	36	1
28	37	1
28	38	1
28	39	1
28	40	1
28	41	1
28	42	1
28	43	1
28	44	1
28	45	1
28	46	1
28	56	1
28	57	1
28	58	1
28	59	1
28	63	1
28	65	1
28	66	1
28	67	1
46	47	1
47	49	1
50	51	1
50	63	1
52	53	1
53	63	1
54	55	1
55	63	1
59	63	1
63	64	1

Relationship between components in product 18

1	2	1
1	8	1
1	12	1
1	29	1
2	3	1
2	4	1
2	5	1
2	6	1
2	8	1
2	10	1
2	11	1
2	12	1
2	13	1
2	29	1
2	70	1
3	12	1
3	13	1
4	5	1
4	6	1
4	7	1
5	6	1
8	9	1
8	28	1
8	29	1
8	59	1
9	10	1
9	29	1
10	15	1
10	16	1
10	17	1
10	18	1
10	19	1
10	20	1
10	21	1
10	24	1
10	25	1
10	26	1
10	28	1
10	29	1
10	48	1
10	49	1
10	50	1
10	51	1
10	52	1
10	53	1
10	54	1
10	55	1
10	56	1
10	57	1
10	60	1
10	62	1
10	63	1
10	64	1
10	65	1
10	66	1
10	67	1
10	68	1
10	69	1
10	71	1
11	12	1
11	13	1
13	14	1
13	29	1
14	15	1
14	16	1
14	29	1
15	17	1
15	18	1
15	29	1
16	17	1
16	18	1
16	29	1
19	20	1
19	65	1
20	57	1
20	66	1
21	27	1
21	28	1
21	59	1
22	24	1
22	27	1
22	28	1
23	24	1
23	27	1
23	28	1
25	26	1
25	27	1
25	28	1
27	28	1
27	63	1
28	29	1
28	58	1
28	62	1
29	30	1
29	31	1
29	32	1
29	33	1
29	34	1
29	35	1
29	36	1
29	37	1
29	38	1
29	39	1
29	40	1
29	41	1
29	42	1
29	43	1
29	44	1
29	45	1
29	46	1
29	47	1
29	57	1
29	59	1
29	60	1
29	61	1
29	65	1
29	67	1
29	68	1
29	69	1
47	48	1
48	50	1
51	52	1
51	65	1
53	54	1
54	65	1
55	56	1
56	65	1
61	65	1
65	66	1

Relationship between components in product 19

1	2	1
1	3	1
1	9	1
1	30	1
3	4	1
3	5	1
3	6	1
3	9	1
3	12	1
3	30	1
3	69	1
5	8	1
6	8	1
7	8	1
7	69	1
8	9	1
9	10	1
9	13	1
9	14	1
9	15	1
9	17	1
9	18	1
9	29	1
9	30	1
9	64	1
9	67	1
9	71	1
9	72	1
10	11	1
10	12	1
10	30	1
11	12	1
12	17	1
12	18	1
12	19	1
12	20	1
12	21	1
12	22	1
12	23	1
12	24	1
12	26	1
12	27	1
12	28	1
12	29	1
12	30	1
12	53	1
12	54	1
12	55	1
12	56	1
12	57	1
12	58	1
12	59	1
12	60	1
12	61	1
12	62	1
12	63	1
12	64	1
12	65	1
12	66	1
12	68	1
12	70	1
12	71	1
12	72	1
13	16	1
13	30	1
13	69	1
14	16	1
14	30	1
14	69	1
15	16	1
15	30	1
15	69	1
17	19	1
17	20	1
17	30	1
18	19	1
18	20	1
18	30	1
21	64	1
22	23	1
22	25	1
22	27	1
22	62	1
23	24	1
23	29	1
25	26	1
25	29	1
27	28	1
27	29	1
29	30	1
29	62	1
30	31	1
30	32	1
30	33	1
30	34	1
30	35	1
30	36	1
30	37	1
30	38	1
30	39	1
30	40	1
30	52	1
30	64	1
30	67	1
30	71	1
30	72	1
31	41	1
31	42	1
31	43	1
31	44	1
31	45	1
31	46	1
31	47	1
31	48	1
31	49	1
31	50	1
31	51	1
52	53	1
53	55	1
56	57	1
56	64	1
58	59	1
59	67	1
60	61	1
61	67	1
64	65	1
66	67	1
67	68	1

Relationship between components in product 20

1	2	1
1	3	1
1	9	1
1	29	1
3	4	1
3	5	1
3	6	1
3	9	1
3	11	1
3	29	1
3	75	1
5	8	1
6	8	1
7	8	1
7	75	1
8	9	1
9	10	1
9	12	1
9	13	1
9	14	1
9	16	1
9	17	1
9	28	1
9	29	1
9	70	1
9	73	1
9	77	1
9	78	1
10	11	1
10	29	1
11	16	1
11	17	1
11	18	1
11	19	1
11	20	1
11	21	1
11	22	1
11	23	1
11	25	1
11	26	1
11	27	1
11	28	1
11	29	1
11	59	1
11	60	1
11	61	1
11	62	1
11	63	1
11	64	1
11	65	1
11	66	1
11	67	1
11	68	1
11	69	1
11	70	1
11	71	1
11	72	1
11	74	1
11	76	1
11	77	1
11	78	1
12	15	1
12	29	1
12	75	1
13	15	1
13	29	1
13	75	1
14	15	1
14	29	1
14	75	1
16	18	1
16	19	1
16	29	1
17	18	1
17	19	1
17	29	1
20	70	1
21	22	1
21	24	1
21	26	1
21	68	1
22	23	1
22	28	1
24	25	1
24	28	1
26	27	1
26	28	1
28	29	1
28	68	1
29	30	1
29	31	1
29	32	1
29	33	1
29	34	1
29	35	1
29	36	1
29	37	1
29	38	1
29	39	1
29	40	1
29	41	1
29	42	1
29	43	1
29	44	1
29	58	1
29	70	1
29	73	1
29	77	1
29	78	1
30	45	1
30	46	1
30	47	1
30	48	1
30	49	1
30	50	1
30	51	1
30	52	1
30	53	1
30	54	1
30	55	1
30	56	1
30	57	1
58	59	1
59	61	1
62	63	1
62	70	1
64	65	1
65	73	1
66	67	1
67	73	1
70	71	1
72	73	1
73	74	1

Relationship between components in product 21

1	2	1
1	3	1
1	9	1
1	31	1
3	4	1
3	5	1
3	6	1
3	10	1
3	13	1
3	31	1
3	77	1
5	8	1
6	8	1
7	8	1
7	77	1
8	9	1
9	11	1
9	14	1
9	15	1
9	16	1
9	18	1
9	19	1
9	30	1
9	31	1
9	72	1
9	75	1
9	79	1
9	80	1
10	31	1
11	12	1
11	13	1
11	31	1
12	13	1
13	18	1
13	19	1
13	20	1
13	21	1
13	22	1
13	23	1
13	24	1
13	25	1
13	27	1
13	28	1
13	29	1
13	30	1
13	31	1
13	61	1
13	62	1
13	63	1
13	64	1
13	65	1
13	66	1
13	67	1
13	68	1
13	69	1
13	70	1
13	71	1
13	72	1
13	73	1
13	74	1
13	76	1
13	78	1
13	79	1
13	80	1
13	81	1
14	17	1
14	31	1
14	77	1
15	17	1
15	31	1
15	77	1
16	17	1
16	31	1
16	77	1
18	20	1
18	21	1
18	31	1
19	20	1
19	21	1
19	31	1
22	72	1
23	24	1
23	26	1
23	28	1
23	70	1
24	25	1
24	30	1
26	27	1
26	30	1
28	29	1
28	30	1
30	31	1
30	70	1
31	32	1
31	33	1
31	34	1
31	35	1
31	36	1
31	37	1
31	38	1
31	39	1
31	40	1
31	41	1
31	42	1
31	43	1
31	44	1
31	45	1
31	46	1
31	60	1
31	72	1
31	75	1
31	79	1
31	80	1
31	81	1
32	47	1
32	48	1
32	49	1
32	50	1
32	51	1
32	52	1
32	53	1
32	54	1
32	55	1
32	56	1
32	57	1
32	58	1
32	59	1
60	61	1
61	63	1
64	65	1
64	72	1
66	67	1
67	75	1
68	69	1
69	75	1
72	73	1
74	75	1
75	76	1

Relationship between components in product 22

1	2	1
1	3	1
1	8	1
1	29	1
2	3	1
2	4	1
2	5	1
2	6	1
2	7	1
3	8	1
3	12	1
3	29	1
3	67	1
4	7	1
5	7	1
6	7	1
6	67	1
7	9	1
8	29	1
9	13	1
9	14	1
9	15	1
9	17	1
9	29	1
9	62	1
9	65	1
9	70	1
9	71	1
10	11	1
10	28	1
10	49	1
10	69	1
11	12	1
11	29	1
12	17	1
12	18	1
12	19	1
12	20	1
12	21	1
12	22	1
12	25	1
12	26	1
12	27	1
12	28	1
12	29	1
12	49	1
12	50	1
12	51	1
12	52	1
12	53	1
12	54	1
12	55	1
12	56	1
12	57	1
12	58	1
12	59	1
12	60	1
12	61	1
12	62	1
12	63	1
12	64	1
12	66	1
12	68	1
12	69	1
12	70	1
12	71	1
13	16	1
13	29	1
13	67	1
14	16	1
14	29	1
14	67	1
15	16	1
15	29	1
15	67	1
17	18	1
17	19	1
17	29	1
20	62	1
21	22	1
21	23	1
21	24	1
21	26	1
21	60	1
22	28	1
23	25	1
23	28	1
24	25	1
24	28	1
26	27	1
26	28	1
28	29	1
28	60	1
29	30	1
29	31	1
29	32	1
29	33	1
29	34	1
29	35	1
29	36	1
29	37	1
29	38	1
29	39	1
29	40	1
29	41	1
29	42	1
29	43	1
29	44	1
29	45	1
29	46	1
29	47	1
29	48	1
29	49	1
29	62	1
29	65	1
29	69	1
29	70	1
29	71	1
49	50	1
49	53	1
50	52	1
54	55	1
54	62	1
56	57	1
57	62	1
58	59	1
59	62	1
59	65	1
62	63	1
64	65	1
65	66	1

Relationship between components in product 23

1	2	1
1	3	1
1	8	1
1	30	1
2	3	1
2	4	1
2	5	1
2	6	1
2	7	1
3	8	1
3	13	1
3	30	1
3	76	1
4	7	1
5	7	1
6	7	1
6	76	1
7	8	1
8	14	1
8	15	1
8	16	1
8	18	1
8	19	1
8	30	1
9	30	1
9	69	1
9	72	1
9	79	1
10	12	1
10	29	1
11	58	1
11	75	1
11	78	1
12	13	1
12	30	1
13	18	1
13	19	1
13	20	1
13	21	1
13	22	1
13	23	1
13	24	1
13	27	1
13	28	1
13	29	1
13	30	1
13	58	1
13	59	1
13	60	1
13	61	1
13	62	1
13	63	1
13	64	1
13	65	1
13	66	1
13	67	1
13	68	1
13	70	1
13	71	1
13	72	1
13	73	1
13	74	1
13	77	1
13	78	1
13	79	1
14	17	1
14	30	1
14	76	1
15	17	1
15	30	1
15	76	1
16	17	1
16	30	1
16	76	1
18	20	1
18	21	1
18	30	1
19	20	1
19	21	1
19	30	1
22	72	1
23	24	1
23	25	1
23	26	1
23	28	1
23	70	1
24	29	1
25	27	1
25	29	1
26	27	1
26	29	1
28	29	1
29	30	1
29	70	1
30	31	1
30	32	1
30	33	1
30	34	1
30	35	1
30	36	1
30	37	1
30	38	1
30	39	1
30	40	1
30	41	1
30	42	1
30	43	1
30	44	1
30	45	1
30	46	1
30	47	1
31	48	1
31	49	1
31	50	1
31	51	1
31	52	1
31	53	1
31	54	1
31	55	1
31	56	1
31	57	1
31	58	1
31	69	1
31	72	1
31	75	1
31	78	1
31	79	1
58	59	1
58	62	1
59	61	1
63	64	1
63	72	1
65	66	1
66	72	1
67	68	1
68	72	1
72	73	1
74	75	1

Relationship between components in product 24

1	2	1
1	7	1
1	29	1
2	3	1
2	4	1
2	7	1
2	11	1
2	29	1
2	70	1
3	6	1
4	6	1
5	6	1
5	70	1
6	8	1
7	10	1
7	28	1
7	29	1
7	67	1
8	12	1
8	13	1
8	14	1
8	16	1
8	17	1
8	21	1
8	73	1
8	74	1
9	55	1
10	11	1
11	12	1
11	13	1
11	14	1
11	16	1
11	17	1
11	18	1
11	19	1
11	20	1
11	21	1
11	22	1
11	23	1
11	26	1
11	27	1
11	28	1
11	29	1
11	55	1
11	56	1
11	57	1
11	58	1
11	59	1
11	60	1
11	61	1
11	62	1
11	63	1
11	64	1
11	65	1
11	66	1
11	67	1
11	68	1
11	69	1
11	71	1
11	72	1
11	73	1
11	74	1
12	15	1
12	29	1
12	70	1
13	15	1
13	29	1
13	70	1
14	15	1
14	29	1
14	70	1
16	18	1
16	19	1
16	29	1
17	18	1
17	19	1
17	29	1
21	29	1
21	68	1
22	23	1
22	28	1
24	26	1
24	28	1
25	26	1
25	28	1
27	28	1
28	29	1
28	69	1
29	30	1
29	31	1
29	32	1
29	33	1
29	34	1
29	35	1
29	36	1
29	37	1
29	38	1
29	39	1
29	40	1
29	41	1
29	42	1
29	43	1
29	44	1
29	45	1
29	46	1
29	47	1
29	48	1
29	49	1
29	50	1
29	51	1
29	52	1
29	53	1
29	54	1
29	55	1
29	67	1
29	72	1
29	73	1
29	74	1
55	56	1
56	58	1
59	60	1
59	67	1
61	62	1
62	67	1
63	64	1
64	67	1
67	68	1

Relationship between components in product 24

1	2	1
1	7	1
1	30	1
2	3	1
2	4	1
2	7	1
2	11	1
2	30	1
2	71	1
3	6	1
4	6	1
5	6	1
5	71	1
6	8	1
7	10	1
7	29	1
7	30	1
7	68	1
8	12	1
8	13	1
8	14	1
8	16	1
8	17	1
8	18	1
8	22	1
8	74	1
8	75	1
9	56	1
10	11	1
11	12	1
11	13	1
11	14	1
11	16	1
11	17	1
11	18	1
11	19	1
11	20	1
11	21	1
11	22	1
11	23	1
11	24	1
11	27	1
11	28	1
11	29	1
11	30	1
11	56	1
11	57	1
11	58	1
11	59	1
11	60	1
11	61	1
11	62	1
11	63	1
11	64	1
11	65	1
11	66	1
11	67	1
11	68	1
11	69	1
11	70	1
11	72	1
11	73	1
11	74	1
11	75	1
12	15	1
12	30	1
12	71	1
13	15	1
13	30	1
13	71	1
14	15	1
14	30	1
14	71	1
16	19	1
16	20	1
16	30	1
17	19	1
17	20	1
17	30	1
18	19	1
18	20	1
18	30	1
22	30	1
22	69	1
23	24	1
23	29	1
25	27	1
25	29	1
26	27	1
26	29	1
28	29	1
29	30	1
29	70	1
30	31	1
30	32	1
30	33	1
30	34	1
30	35	1
30	36	1
30	37	1
30	38	1
30	39	1
30	40	1
30	41	1
30	42	1
30	43	1
30	44	1
30	45	1
30	46	1
30	47	1
30	48	1
30	49	1
30	50	1
30	51	1
30	52	1
30	53	1
30	54	1
30	55	1
30	56	1
30	68	1
30	73	1
30	74	1
30	75	1
56	57	1
57	59	1
60	61	1
60	68	1
62	63	1
63	68	1
64	65	1
65	68	1
68	69	1
